# Self-Organizing Proteinoid–Actin Networks: Structure and Voltage Dynamics

**DOI:** 10.1021/acsomega.5c01141

**Published:** 2025-05-05

**Authors:** Panagiotis Mougkogiannis, Andrew Adamatzky

**Affiliations:** Unconventional Computing Laboratory, University of the West of England, Bristol BS16 1QY, U.K.

## Abstract

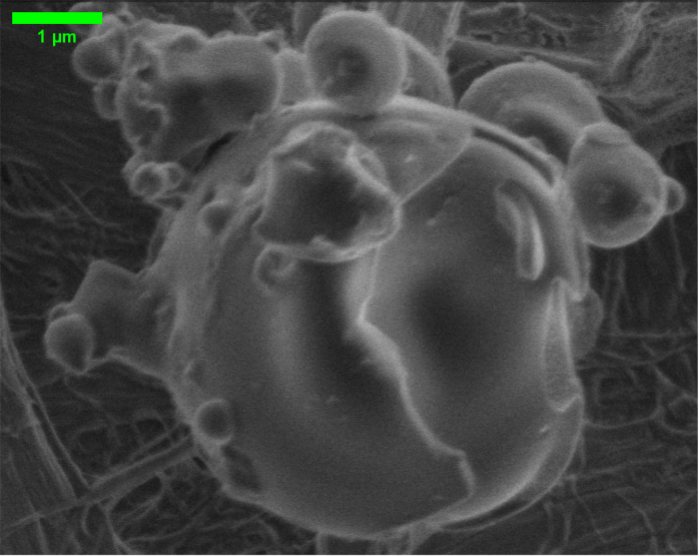

Proteinoids are thermal proteins produced by heating amino acids to their melting point and initiation of polymerization to produce polymeric chains. Proteinoids swell in aqueous solution forming hollow microspheres, usually filled with aqueous solution. The microspheres produce spikes of electrical potential similar to the action potentials of living neurons. The cytoskeletal protein actin is known in its filamentous form as F-actin. Filaments are organized in a double helix structure consisting of polymerized globular actin monomers. Actin is a protein that is abundantly expressed in all eukaryotic cells and plays a crucial role in cellular functions by forming an intracellular scaffold, actuators, and pathways for information transfer and processing. We produce and study proteinoid-actin networks as physical models of primitive neurons. We look at their structure and electrical dynamics. We use scanning electron microscopy and multichannel electrical recordings to study microsphere assemblies. They have distinct surface features, including ion channel-like pores. The proteinoid–actin mixture exhibits enhanced electrical properties compared to its individual components. Its conductivity (σ = 4.68 × 10^–4^ S/cm) is higher than those of both pure actin (σ = 1.23 × 10^–4^ S/cm) and pure proteinoid (σ = 2.45 × 10^–4^ S/cm). The increased conductivity and new oscillatory patterns suggest a synergy. They indicate a synergy between the proteinoid and actin components in the mixture. Multichannel analysis reveals type I regular spiking in proteinoid networks (Δ*V* ≈ 50 mV, τ = 52.4 s), type II excitability in actin (*V*_max_ ≈ 40 mV), and bistable dynamics in the mixture. These findings suggest that proteinoid–actin complexes can form primitive bioelectrical systems. This might lead to the better understanding of the evolution of the primordial neural system.

## Introduction

Protein mechanics has revealed fascinating insights into living systems at various scales.^1−4^ Protein mechanics is vital to many cellular processes.^5^ It ranges from the dynamics of individual amino acids to the motion of complex biological structures.^6,7^ One such area of interest is the actin cytoskeleton.^8−11^ It is a dynamic network of protein filaments.^9^ It generates and transmits mechanical forces within cells. The actin cytoskeleton is not a static scaffold.^12^ It is a highly adaptive, responsive structure. Its networks are constantly changing and reorganizing.^12^

The actin filaments in the cytoskeleton can polymerize and depolymerize.^13^ Dynamic reorganization of this cytoskeleton is essential for cellular processes such as cell division, neural plasticity, wound healing, and metastasis. This happens in a coordinated way, driven by ATP hydrolysis.^14,15^ This behavior lets the actin network quickly change in response to internal and external signals. It enables cell migration, organelle transport, and tissue morphogenesis.^6^ The actin cytoskeleton is a key part of cell structure. It works within a complex web of molecular interactions and regulatory pathways.^16^ It does not function as an isolated structure. It is part of a larger system of protein networks and regulatory pathways.^17,18^ These are interconnected. These include intermediate filaments, like vimentin. They are key to cell shape and strength.^19^ Also, the actin network is linked to the microtubule cytoskeleton.^18,20^ The two systems work together to coordinate transport and organization within the cell.^6^

New advances in microscopy and modeling have given us insights into the actin cytoskeleton and its protein networks. These studies show a complex link between the cytoskeleton’s structure and function.^7,18,21,22^ Its mechanical properties are tied to its electrical and signaling abilities. For example, a study of actin-based motility has shown that the coordinated growth and shrinkage of actin filaments can generate powerful forces. These forces are essential for cell migration and the transport of organelles.^9^ Moreover, the actin cytoskeleton is closely linked to the cell membrane. The two systems work together to sense and respond to mechanical signals from the outside environment.^12^ Researchers are studying the complex interplay between the actin cytoskeleton and its protein networks. It involves their structural, mechanical, and electrical properties.^23,24^

To better understand the principles of neural cellular emergence, organization, and function,^25,26^ we must explore how protein-based networks affect their electrical properties.^27−32^ This research studies the self-organizing properties and voltage dynamics of proteinoid-actin networks. It aims to find parallels with primitive cellular behavior.^33^ Proteinoids, thermal polymers of amino acids, are considered to be physical models of protoneurons.^25,28,34^ They self-assemble into microspheres with membrane-like properties. We aim to explore the hybrid networks’ properties by combining two distinct protein systems.^29,31^ We will focus on their self-organization and electrical dynamics. We hypothesize that proteinoid-actin networks have unique properties. Their self-organization and voltage dynamics differ from their individual parts. They may mimic primitive cellular behaviors. This research addresses several key questions:

(1) How do proteinoids and actin interact to form hybrid networks? What are the networks’ structural characteristics?
(2) Do these networks show spontaneous voltage changes? If so, how do they compare to those in pure proteinoid and pure actin systems?
(3) What are the mechanisms behind the self-organization and electrical behavior of these hybrid networks?
(4) What do these findings mean for studying primitive cells? And for designing synthetic cells?

This study will use both experimental and computational methods. It will investigate the formation, structure, and voltage dynamics of proteinoid-actin networks. This study aims to synthesize and characterize proteinoid-actin complexes. It will focus on their network formation and stability. This research aim to find new behaviors in cells. It will analyze voltage changes across multiple channels. It will also compare hybrid networks to their pure components: proteinoid and actin systems. These insights may help us understand complex cellular functions. This work could link protoneural systems to modern neurons. It may provide new views on how biological complexity evolved. It fills a gap in our knowledge of voltage dynamics in proteinoid-based systems and hybrid proteinoid-protein networks. Previous studies have explored the self-assembly of proteinoids and actin networks.^23,24^ But, the electrical properties of their combined systems are largely unexplored. This work could help us understand primitive cellular processes. It may impact bioengineering, biomaterials, and synthetic cellular systems. Furthermore, it may provide insights into the role of bioelectric phenomena in the origin of life.^35^

Our study is the first to look at proteinoid microspheres and actin networks together. Previous research focused on them as distinct models for protocells and for self-organization.^36−41^ We go beyond earlier studies that looked at proteinoids’ structure or actin filaments’ mechanics. Instead, we focus on the electrical properties that emerge from their interactions. This approach shows how proto-biomolecular systems may have gained signaling abilities. This study stands out because we combine microscale electrical measurements with real-time visualization. This approach reveals new dynamics between synthetic and biological polymers. Traditional methods overlook these details. Our hybrid system shows how early evolutionary precursors changed into neural signaling networks. Our approach is different from past studies. Instead of using only synthetic or only biological parts, we combine both. This allows for a unique exploration.

## Experimental Section

Commercial rabbit skeletal muscle actin (Cytoskeleton, Inc.) was combined with l-phenylalanine and l-glutamic acid (Sigma-Aldrich) to make proteinoid–actin composites. Proteinoid synthesis followed thermal polycondensation at *T* = 180 °C under reflux for *t* = 30 min. Actin (ω = 1% w/w) was added during polymerization. The resulting materials were lyophilized and characterized using SEM (Quanta 650).

For all experiments, precise concentrations were maintained to ensure reproducibility. The proteinoid solution was made at 15 mg/mL in aqueous solution. G-actin was used at a final concentration of 5 μM (0.21 mg/mL) in G-buffer. To form the composite, the solutions were mixed in a 3:1 volume ratio (proteinoid:actin). They were allowed to stir for 30 min at room temperature (23 ± 2^◦^C). This ratio was found to create the best network formation and electrical activity.

**Figure 1:**
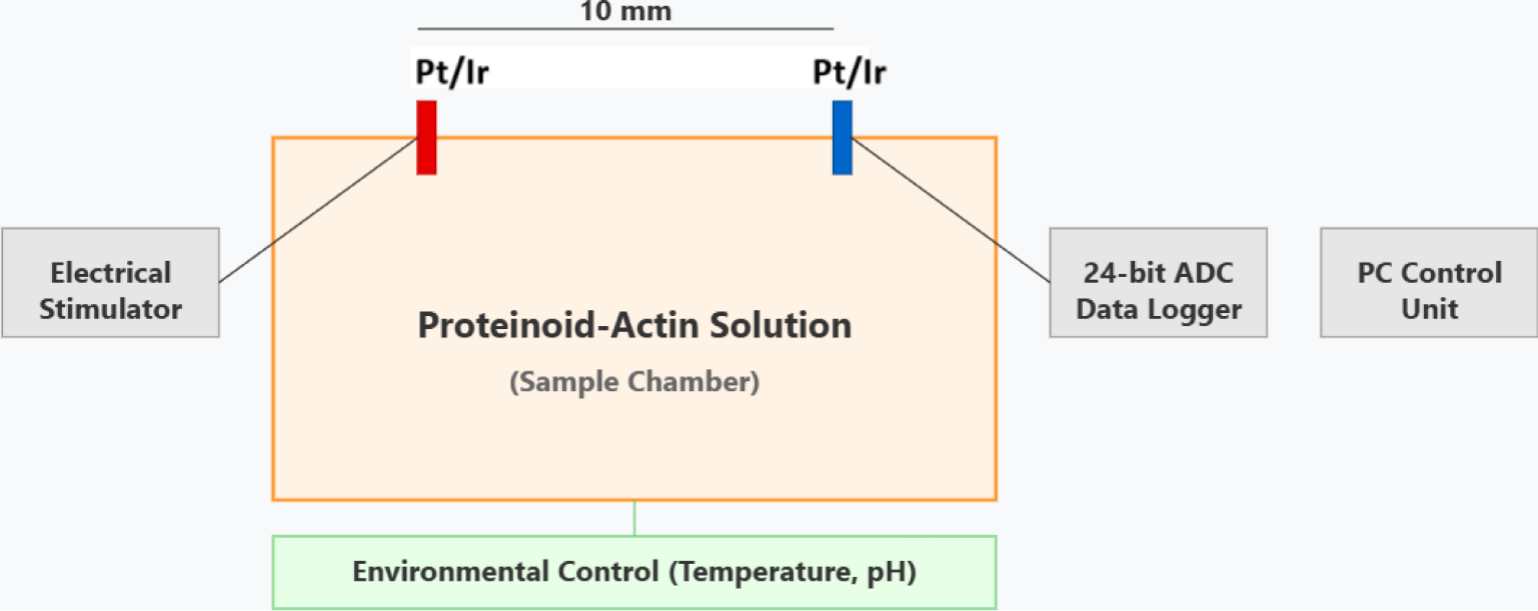
Schematic representation of the experimental setup for proteinoid–actin electrical measurements. The configuration utilized Pt/Ir electrodes. They are 10 mm apart. They interface with the proteinoid–actin solution in a temperature-controlled chamber. Signal generation and acquisition are achieved through an electrical stimulator and a 24-bit ADC data logger (δ*V* = 0.97 μV resolution) connected to a PC control unit. Customized sensors continuously monitor environmental parameters (*T* = 25 ± 0.1 °C, pH 7.4 ± 0.01). This ensures stable conditions for measuring spontaneous electrical activity.

Electrical measurements utilized custom Pt–Ir-coated steel electrodes (Spes Medica Srl.) positioned *d* = 10 mm apart within the sample chamber (Figure 1). We recorded voltage using a PicoTechnology ADC-24 data logger. We also took complementary potentiometric measurements (Δ*V*_range_ = ± 5 V) with an Ossila T2006A system. The measurement cell kept conditions (*T* = 25 ± 0.1 °C, pH 7.4 ± 0.01) during data acquisition. This allowed precise monitoring of spontaneous electrical activity across the proteinoid-actin network.

We recorded voltage using a PicoTechnology ADC-24 data logger in differential mode, where the measured voltage was calculated as *V*_diff_ = *V*_A_ – *V*_R_, with *V*_A_ and *V*_R_ representing the potentials at the active electrode and reference electrodes, respectively. Data was collected at a sampling rate of 2.5 Hz (Δ*t* = 0.4 s). Electrochemical characterization was done using cyclic voltammetry (CV). We used a T20064 Potentiostat (Ossila, Sheffield, UK) for the experiments. We measured complex impedance (*Z*/*Z*”) using a PalmSens4 electrochemical interface (PalmSens ALVATEK, UK). The electrochemical impedance spectroscopy (EIS) measurements used a fixed scan mode. The DC potential (*E*_dc_) was set at 0.2 V, and the AC perturbation amplitude (*E*_ac_) was 0.01 V. The frequency range went from 0.0001 Hz to 100 kHz, covering 198 points per decade. Measurements were performed versus the open circuit potential (OCP) with a maximum OCP measuring time (*t*_Max,OCP_) of 1.0 s and a stability criterion of 0.0 mV/s. All experiments were carried out at room temperature (25 ± 1 °C) using a standard three-electrode configuration. It has a gold screen-printed working electrode, a platinum counter electrode, and an iridium quasi-reference electrode. The electrochemical cell was set up in a Faraday cage to minimize external electromagnetic interference.

## Results and Discussion

### Self-Assembly and Structural Dynamics of Proteinoid–Actin Filament Networks

**Figure 2:**
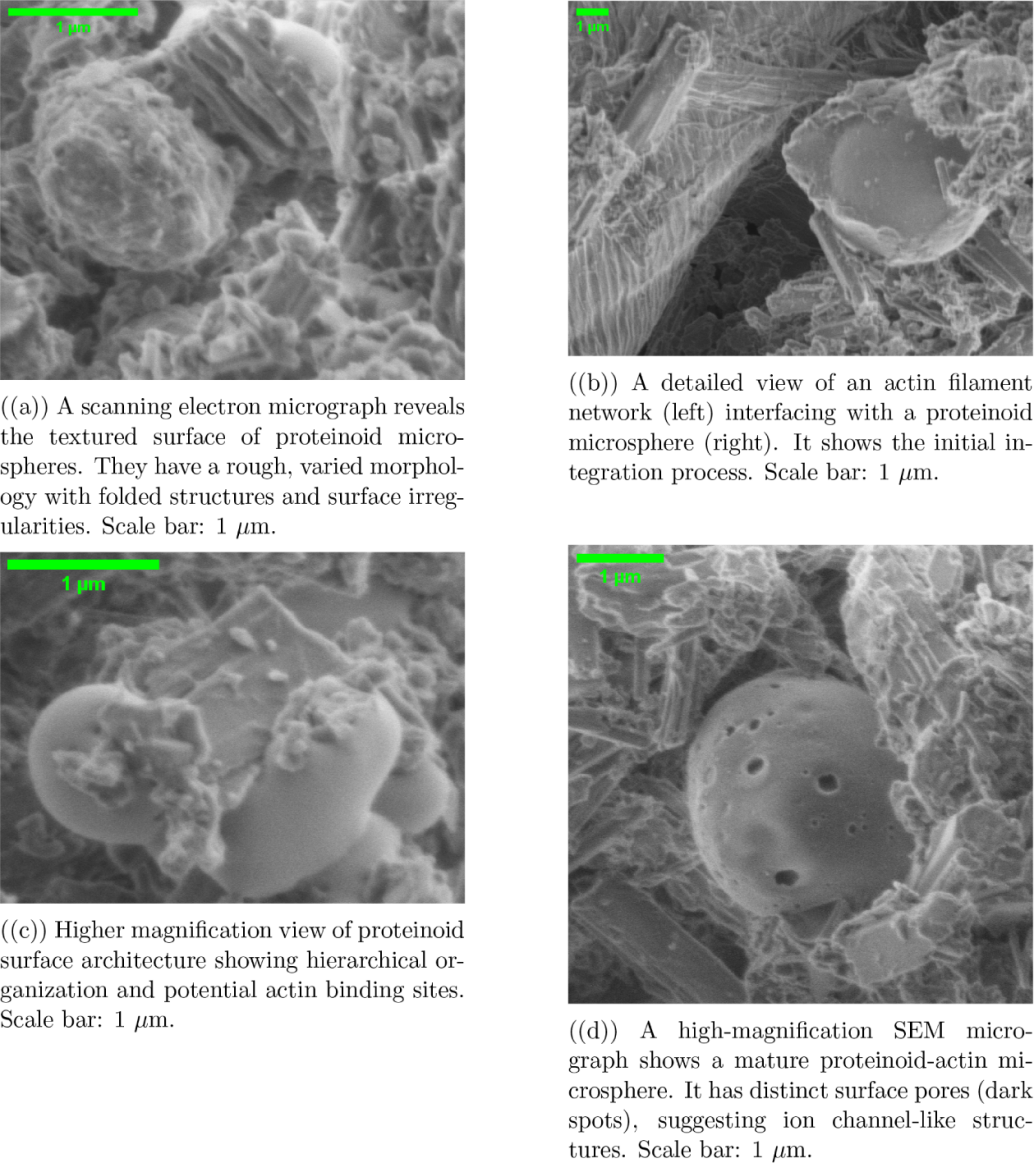
SEM analysis shows actin filaments progressively incorporating into proteinoid microspheres. The sequence shows a hierarchy from initial network formation (b) to mature microspheres (a) with integrated actin and developed surface features (c, d). The absence of conductive coating allows direct visualization of the native surface features.

Scanning electron microscopy showed the actin filaments integrated with proteinoid microspheres. The morphology can be seen in the Supporting Information (Figure S10). It also revealed their unique features (Figure 2). High-res imaging of uncoated samples, done under high vacuum (1.45–1.75 × 10^–5^ Torr), provided detailed surface data without coating artifacts.

The microsphere populations exhibited diverse dimensions, ranging from 1.639 to 5.539 μm in diameter. Mature proteinoid–actin microspheres had distinct surface pores. They averaged 0.283 μm in diameter (Figure 2d). We imaged them at 25,000× magnification using an Everhart–Thornley Detector at 1.10 kV. These structures suggest potential ion channel-like features integrated within the microsphere architecture.

The interface between actin filaments and proteinoid microspheres was most evident in the intermediate stages of formation. Actin filaments, 0.9–1.139 μm long and 0.047–0.110 μm thick, formed complex networks around and within the microspheres (Figure 2b,c). Higher magnification imaging (41,771×) revealed internal filaments. They measured 1.110 × 0.899 μm. This suggests that actin networks were successfully incorporated into the proteinoid matrix.

The composite system’s hierarchy was clear at 45,552× magnification (Figure 2a). We could see the surface texture and filament integration sites. This structural hierarchy, seen at multiple length scales, shows a systematic process of actin-proteinoid assembly and organization.

The high-magnification analysis revealed unique openings in individual microspheres (Figure 2d). This suggests a new feature in the proteinoid-actin interface. These pores, precisely measured at 0.283 μm in diameter, appear to be associated with actin filament attachment points. The integration process may form these pores. Actin filaments penetrate and anchor within the proteinoid matrix.

The system studied comprises proteinoid microspheres, proteinoid crystals, and actin networks. Their interface with actin filaments shows this (Figure 2b). This is important. It shows a direct link between ordered proteinoid crystals and the actin network. The organized interface between the proteinoid regions and protein filaments suggests paths for charge transport and electrical coupling in the hybrid system.

These features—the pore formation and crystalline-filament interfaces—may explain the electrical activity of these composites. The crystalline proteinoid domains and conductive protein filaments are arranged regularly. This could create regions that support charge separation and transport. It may explain the system’s electrophysiological properties.

**Figure 3:**
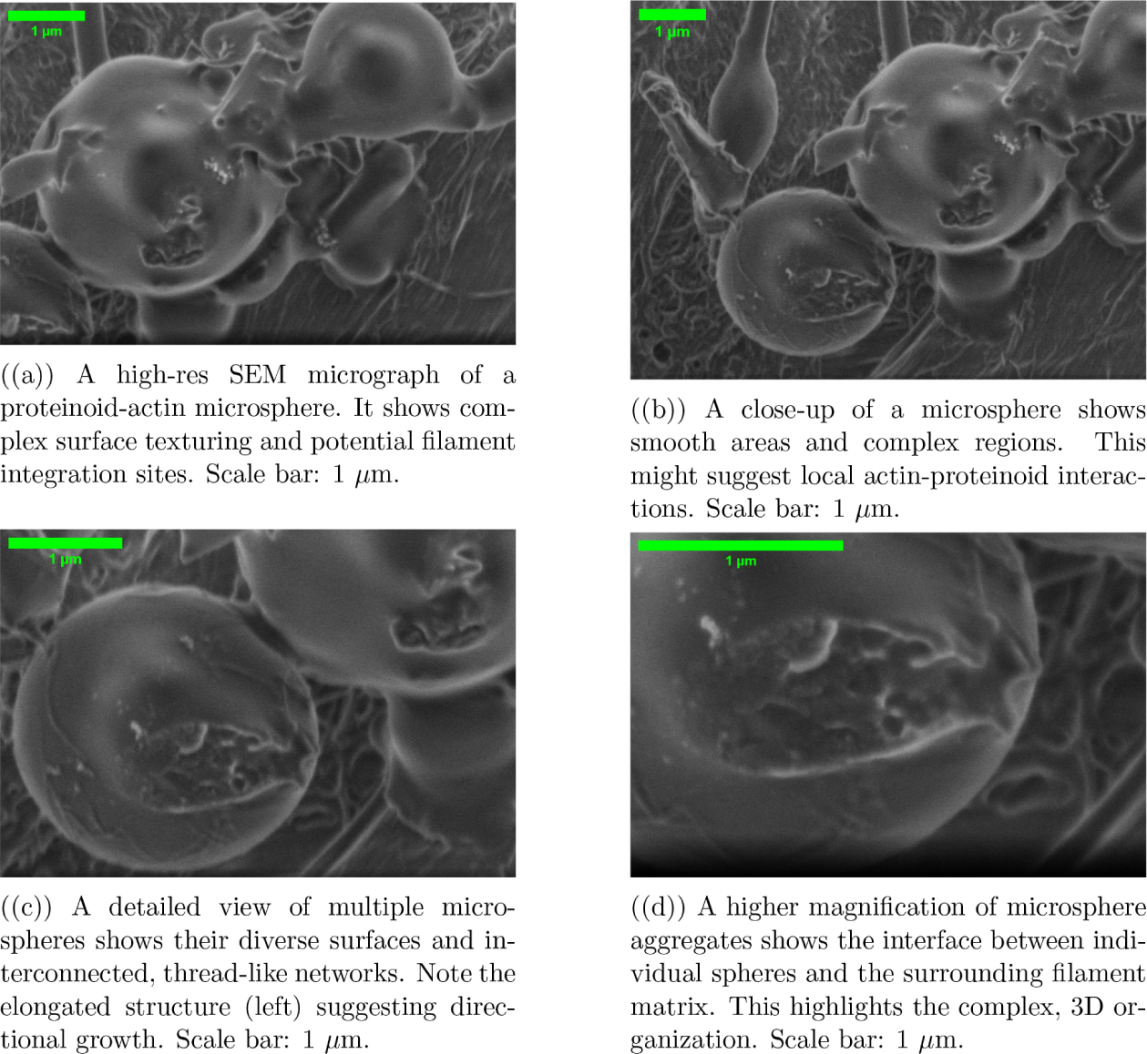
Scanning electron microscopy of proteinoid–actin composites reveals a multiscale, hierarchical organization. We acquired images using an ETD detector under high vacuum (3.29–3.52 × 10^–6^ Torr). We used low-voltage beam parameters (1.50 kV) to minimize charging effects on these uncoated specimens. The sequence shows the complex link between spherical proteinoid structures and their filamentous networks.

Figure 3a was taken at 30,000× magnification. The settings were 1.50 kV, a spot size of 1.7, and a 4.5 mm working distance. The chamber pressure was maintained at 3.52 × 10^–6^ Torr with a horizontal field width (HFW) of 6.91 μm. Figure 2b was imaged at 23,133× magnification. It used the same beam conditions (1.50 kV, spot size 1.7) and working distance (4.5 mm). The chamber pressure was 3.29 × 10^–6^ Torr and the HFW was 8.96 μm. Figure 2a,d was captured under similar low-voltage conditions to reduce charging effects on the uncoated specimens.

SEM analysis suggests that actin may polymerize in proteinoid microspheres (Figure 3b,c). The surface and internal texturing visible through the microsphere boundaries suggest potential actin filament organization inside the proteinoid matrix. Several morphological features support this hypothesis. First, distinct surface deformations may be actin-proteinoid interaction sites. Second, filamentous structures appear to emerge from or penetrate the microspheres. Third, visible texture gradients suggest internal organization. SEM imaging is surface-specific, limiting direct visualization of internal actin polymerization. However, the observed patterns suggest a dynamic interaction. It likely involves internal actin polymerization, beyond just surface attachment, with the proteinoid matrix. Such internal organization could greatly improve the system’s mechanical and electrical properties.

The varied microsphere sizes and surface pores align with the published data on molecules’ organization and dynamics on living cell surfaces.^42−46^ Lipid rafts are areas in the plasma membrane with different compositions. Studies show that their dynamics, distribution, and clustering are key to understanding cell behavior.^47−49^ The interaction between actin and proteinoid–microspheres seems crucial. It affects the formation and stability of the microspheres. It is hypothesized that the proteinoid component provides a framework. The actin component contributes to the microspheres’ dynamic properties. The interaction between proteinoid and actin might explain the differences in microsphere sizes and the creation of surface pores.

The ion channel-like features in the proteinoid-actin microspheres raise questions about their possible functions.

- Could these microspheres be selective filters?
- Can they regulate the passage of specific ions or molecules?

We need more research to understand these ion channel-like features.^50,51^ This includes their selectivity, gating mechanisms, and role in the microspheres’ function. These aspects could reveal uses for proteinoid-actin microspheres. They may help in drug delivery, biosensors, and artificial cells. We must explore these microspheres in different environments and stimuli. This is key to unlocking their full potential.^52−55^

Also, a key point arises from the microsphere size analysis (Figure 3c). Two microspheres have diameters of 3.326 and 2.40 μm. They are connected by a budding region 0.874-μm long. This asymmetric binary structure is remarkably reminiscent of primitive cell division mechanisms,^56^ potentially providing insights into the early evolution of cellular processes.^57^ The size of the parent and daughter microspheres, along with a defined budding zone, suggests that these proteinoid–actin systems may self-reproduce.^58^ This finding has major implications for origin-of-life studies.^59^ It shows that simple, proteinoid-based structures can have biological-like processes.^60^ The exact sizes of the microspheres and their connecting region prove a nonrandom, structured growth. It resembles primitive cellular reproduction. Such spontaneous organization and reproduction in a simple chemical system offers insights into how early prebiotic structures might have developed self-replication methods before modern cellular machinery emerged.

**Figure 4:**
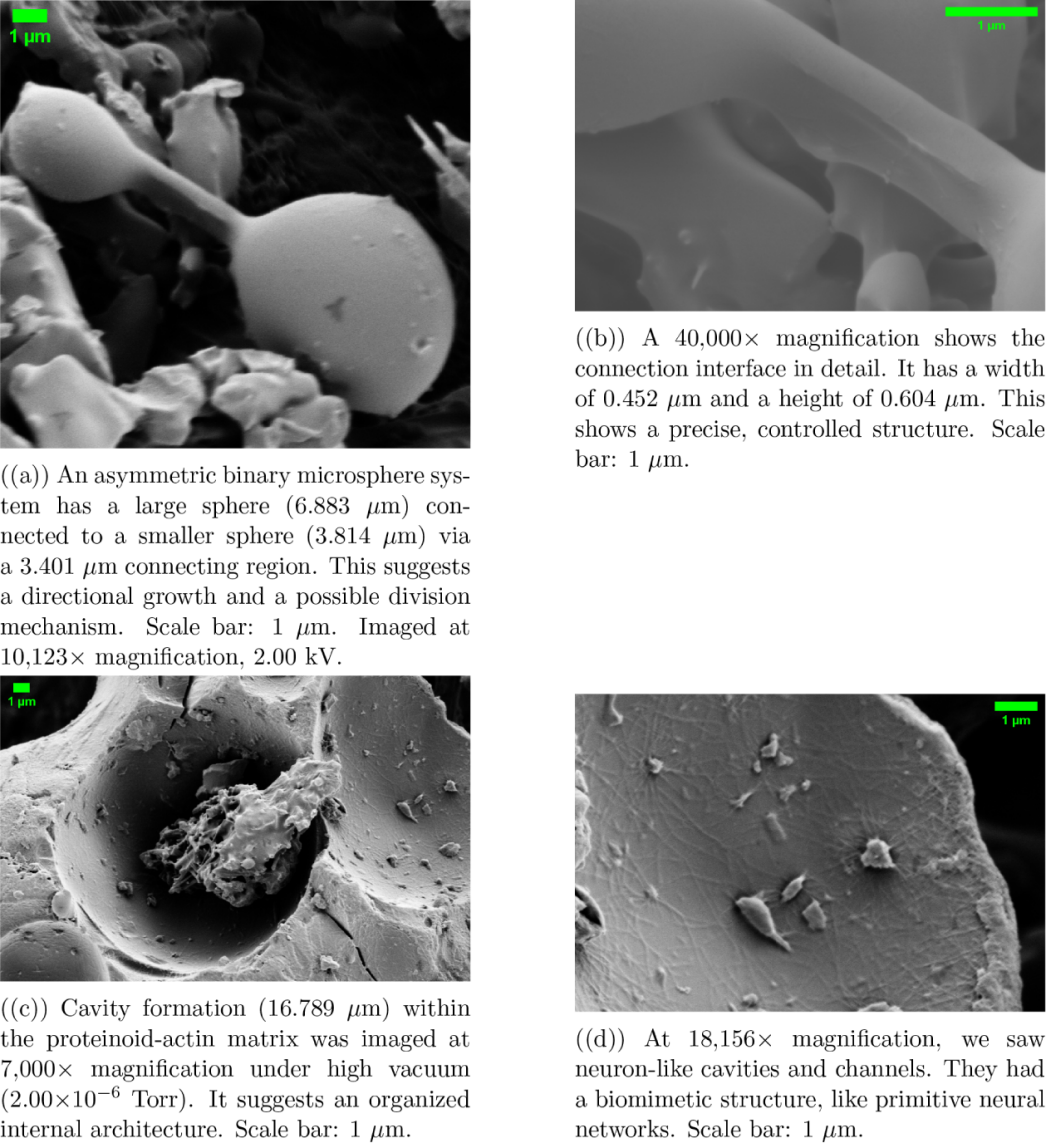
Scanning electron microscopy analysis of proteinoid–actin structural organization revealing hierarchical complexity. Images acquired using an ETD detector at 2.00 kV and spot size 3.0, under consistent high-vacuum conditions (1.43–2.00 × 10^–6^ Torr). The sequence shows many scales of organization. It goes from molecular connections to large biomimetic structures.

Further analysis revealed a remarkable hierarchical structure (Figure 4). The system exhibits precisely defined dimensional characteristics across multiple scales. A striking feature is the asymmetric binary structure (Figure 4a). A larger microsphere (6.883 μm) connects to a smaller one (3.814 μm) via a 3.401 μm bridge. Higher magnification analysis of these connection interfaces (Figure 4b) reveals precise dimensional control, with consistent width (0.452 μm) and height (0.604 μm) measurements.

Of particular significance is the emergence of large cavities (Figure 4c), up to 16.789 μm. They interconnect through a network of channels. These cavities become more specialized. They then resemble primitive neurons (Figure 4d). The dimensional hierarchy, from nanoscale molecules to microscale cavities, suggests ways for signal propagation and processing.

The implications for information processing are multifaceted. (i) The precise control of connecting regions could enable controlled signal transmission. (ii) The hierarchical cavities may allow for localized processing. (iii) The neuron-like channel networks suggest routes for signal integration and distribution. These features, emerging in a proteinoid-actin system, hint at how primitive information processing architectures might have developed before specialized cellular machinery evolved. The same size ratios across scales (0.452 μm connections to 16.789 μm cavities) suggest an organization principle. It may enable coordinated signal processing and transmission.

A detailed study shows multiple structural levels in the proteinoid-actin composite (Table 1). The surface architecture is very diverse. It shifts between smooth and textured areas. Crystalline formations suggest ordered molecular arrangements. These distinct surface features likely serve as active interface zones for actin-proteinoid interactions.

**Table 1:** Morphological Characterization of Proteinoid–Actin Composite Structures

Structural Feature	Morphological Observations	Potential Functions/Implications
Surface texture	Heterogeneous surface with distinct regions of smooth and textured areas; presence of crystalline-like domains	Interface zones for actin–proteinoid interactions; potential sites for molecular recognition^61−64^
Internal organization	Complex network of cavities and channels; visible internal structuring; layered architecture	Compartmentalization; potential for molecular transport and signal propagation^65−67^
Actin integration sites	Distinct surface deformations; filamentous networks emerging from and penetrating microspheres	Active integration points; structural reinforcement; possible mechanosensing regions^68−71^
Interface connections	Bridge-like structures between microspheres; defined connection regions with specific morphology	Communication pathways; material transport; structural integration^72−75^
Growth patterns	Asymmetric binary structures; budding-like formations; directional growth features	Self-reproduction capabilities; controlled growth mechanisms^76−79^
Membrane features	Pore-like structures; surface invaginations; selective permeability indicators	Ion channel-like functionality; controlled molecular exchange^80−83^
Neural-like structures	Channel networks; branching patterns; interconnected cavities	Primitive information-processing architecture; signal propagation pathways^84−87^

Most notably, the internal architecture exhibits sophisticated organization patterns. The linked cavities and channels, plus a layered structure, suggest possible compartmentalization mechanisms. They are similar to primitive cellular organization. Actin integration sites have specific surface deformations and filamentous networks. They may be active zones of structural reinforcement and mechanosensing.

The connections between microspheres are important because they are an indication of material transmission, including ionic flow, between the microspheres. They have unique shapes. These bridge-like structures may help with intersphere communication and transport. They might enable coordinated behavior across the composite system. The growth patterns, like asymmetric binaries and budding, suggest a self-reproduction ability. They may reflect basic principles of protocellular development.

The membrane has intriguing features, like pore-like structures and surface invaginations. They suggest it may function like an ion channel. These features, and the neural-like architectures, suggest a primitive ability to process information. These architectures have complex channel networks and branching patterns. Such a complexity in a synthetic system may explain how early life evolved from simple chemicals.

The distinctive brush-like morphology of actin filaments arises from their fundamental molecular organization. F-actin (filamentous actin) forms when G-actin (globular actin) monomers polymerize into a double-stranded helix. This assembly is hierarchical. It is driven by ATP-dependent polymerization. G-actin monomers (∼5.5 nm in diameter) associate end-to-end. This forms two intertwined strands with a helical pitch of 37 nm. The observed width of ∼92 nm is from bundles of F-actin filaments. They aggregate laterally through electrostatic and hydrophobic interactions. This bundling is enhanced by divalent cations (primarily Mg^2+^ and Ca^2+^), the inherent polarity of actin filaments (barbed (+) and pointed (−) ends), and cross-linking proteins naturally present in the actin preparation. The brush-like architecture comes from the actin polymerization. It is dynamic. Filaments can branch and form networks, especially at the growing (barbed) ends. This creates the hierarchical structures seen in the SEM image (Figure S10).

### Spontaneous Action Potential-Like Spikes in Proteinoid–Actin Hybrid Systems

**Figure 5:**
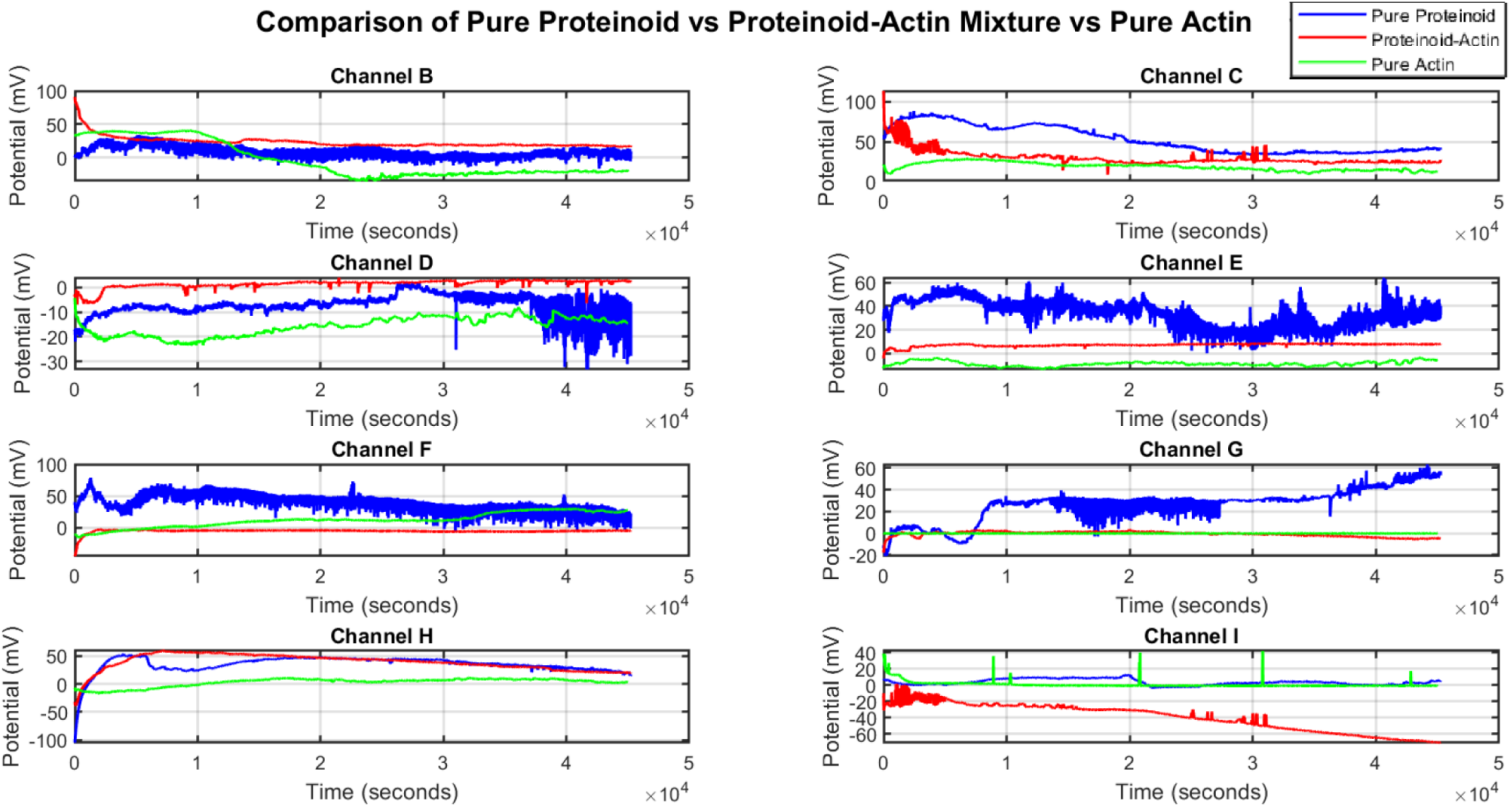
We conducted a multichannel analysis of spontaneous electrical potential (Δψ) oscillations. We compared pure proteinoid, a proteinoid–actin complex, and pure actin systems. The measurement lasted for 5 × 10^4^ s. Membrane potential measurements across eight channels (B–I) reveal distinct dynamics. The proteinoid–actin complex shows oscillatory patterns with Δψ amplitudes of 20–60 mV (±5 mV) in channels C and E. In contrast, channel I shows a monotonic decrease in Δψ from 0 to −60 mV. Pure proteinoid has higher baseline potentials () and stronger oscillations (δψ/δ*t*). This is especially true in channels E (Δψ_max_ ≈ 60 mV) and G (Δψ_max_ ≈ 40 mV). The proteinoid–actin complex (P–A) shows unique, nonlinear behaviors. In channels B and H, τ_decay_ ≈ 10^4^ s. In channel G, it has a biphasic response with initial oscillations (*t* ≈ 10^3^ s), followed by steady-state equilibration. Pure actin maintains quasi-steady-state potentials (ψ_ss_) with minimal fluctuations (σ_ψ_ < 5 mV) across all channels. This suggests that the oscillations come from specific proteinoid–actin interactions, not from the properties of the individual components. The multichannel potential dynamics suggest possible ion transport mechanisms. They indicate membrane-like traits of the proteinoid–actin complex. This aligns with self-assembly and molecular recognition.

The analysis showed different electrical behaviors in proteinoid, actin, and their composites (Figure 5). The proteinoid–actin complex showed unique oscillatory patterns. Channels C and E had potential fluctuations (Δψ) of 20–60 mV (±5 mV). Notably, channel I showed hyperpolarization. The membrane potential fell to −60 mV.

Pure proteinoid networks had higher baseline potentials . They also showed more oscillatory behavior (δψ/δ*t*). This was especially true in channels E and G. There, maximum potential changes (Δψ_*max*_) were about 60 mV and 40 mV, respectively. The proteinoid–actin complex showed complex dynamics over time. Channels B and H had long decay times (τ_decay_ ≈ 10^4^ s). Channel G showed biphasic behavior, with oscillations at *t* ≈ 10^3^ s, followed by steady-state equilibration.

Pure actin systems showed stable quasi-steady-state potentials (ψ_*ss*_). The fluctuations were minimal, with σ_ψ_ < 5 mV across all channels. This indicates that actin mainly acts as a conductor. The oscillations mainly come from proteinoid microspheres. Actin likely serves as a network that helps transport charge through the system. This suggests that proteinoid structures drive the dynamic electrical behavior. Actin provides the pathways for charge propagation. It is not due to the properties of either component alone. The multichannel potential dynamics support organized ion transport. They also suggest membrane-like traits in the proteinoid-actin complex. This is consistent with its ability for self-assembly and molecular recognition.

Statistical analysis of the Glu–Phe proteinoid networks’ spontaneous electrical activity revealed distinct patterns in its amplitude and timing (Figure S1 and Table S1). The electrical measurements were recorded across multiple electrodes (channels B–I) and each channel showed distinct characteristics in terms of amplitude. Channel F had robust oscillations (median = 38.42 ± 7.24 mV). Channel I had more subtle fluctuations (median = 2.47 ± 1.14 mV). This spatial heterogeneity in amplitude suggests localized domains of electrical activity within the network. The temporal characteristics further support organized but heterogeneous behavior. Channels B–H had consistent oscillatory patterns. Their periods ranged from 1100 to 1600 s, with stable standard deviations (σ ≈ 250–320 s). In contrast, channel I had different dynamics. It had longer periods (median = 1580.55 s) and a wider distribution (σ = 1427.39 s). The temporal signatures and amplitude distribution show many ways to create oscillations in the proteinoid network. High-amplitude events occurred at irregular intervals across channels. This was especially evident in channel H (max = 68.63 mV). It suggests occasional burst-like activity. Table S1 shows complex behavior in the proteinoid networks. It lists variations in amplitude and period across channels. This may reflect information-processing abilities.

The spontaneous electrical activity of Glu-Phe proteinoid networks and pure actin systems showed distinct patterns of membrane potential fluctuations. In proteinoid networks (Table S1), the amplitude analysis showed pronounced channel-specific behavior. Channel F had the highest median amplitude (*V*_median_ = 38.42 mV, σ = 7.24 mV). Channels B and E followed, with *V*_median_ ≈ 27 mV. The temporal characteristics were consistent across Channels B–H. The periods ranged from 1111.75 to 1321.80 s, with a standard deviation of σ ≈ 250–320 s. Channel I displayed distinct behavior with broader temporal distribution (σ = 1427.39 s).

Pure actin networks (Table S2) exhibited different electrical characteristics. Channel B showed exceptionally high-amplitude activity (*V*_mean_ = 75.12 mV, σ = 1.25 mV) with stable periodicity (τ = 7225.05 s). Most channels (C–H, excluding G) demonstrated lower amplitude responses (*V*_mean_ = 3–4 mV) but with substantial temporal variability. Channel I showed intermediate amplitude (*V*_mean_ = 26.29 mV, σ = 14.09 mV) and notably extended periods (τ_mean_ = 7121.08 s, σ = 4742.22 s).

The analysis shows that proteinoid networks create more pronounced electrical oscillations. They oscillations are uniform and organized, with consistent periods and moderate amplitudes across multiple channels. In contrast, pure actin networks show less organized oscillatory activity with high variability of parameters. They have extreme amplitude variations between channels and much longer periods. These differences suggest that proteinoid structures may be better than pure actin networks. They may provide more stability and control to membrane potential dynamics. This might be due to organized molecular self-assembly and controlled ion transport.

The analysis of the proteinoid–actin mixture shows that it has distinct electrical behaviors, unlike its individual components. In the mixture (Table S3 and Figure S3), channel C has the highest mean amplitude (*V*_mean_ = 27.66 mV, σ = 8.66 mV). This differs from pure proteinoid networks, where channel F dominates (*V*_median_ = 38.42 mV, σ = 7.24 mV). This suggests a reorganization of electrical activity patterns upon mixing.

The temporal characteristics demonstrate interesting transitions. Pure actin networks show long periods in channel B (τ_mean_ = 7225.05 s) and channel I (τ_mean_ = 7121.08 s). The mixture has more moderate periodicities, with channel E showing the longest periods (τ_max_ = 12441.45 s). Figure S3b shows this temporal distribution. Most channels had periods between 1000 and 2000 s. This suggests a stabilizing effect of the proteinoid–actin interaction.

The mixture’s amplitude distributions (Figure S3a) show unique patterns. Channel C has high median values and high variability. This contrasts with pure proteinoid networks. They have uniform amplitude distributions across channels (σ ≈ 3–13 mV). In pure actin networks, amplitudes vary greatly between channels (*V*_max_ = 76 mV in channel B versus *V*_mean_ ≈ 3–4 mV in most others).

The analysis reveals unique, channel-specific behaviors across the three systems. Pure actin shows a high amplitude in channel B (*V*_mean_ ≈ 75 mV) and long durations (τ ≈ 7000 s). The proteinoid–actin mixture has more moderate, distributed responses, peaking in Channel C (*V*_mean_ ≈ 28 mV). The mixture’s electrical behavior suggests a synergy between the components. There was a change in amplitude and timing across channels. Notably, channel B’s dominant activity was reduced to moderate levels. Also, the periodic responses became more uniform (τ ≈ 1000–2500 s).

The stats imply that the proteinoid-actin mix has new, unique electrical properties. They are different from either component alone. The mixture shows varied amplitudes and complex patterns. This suggests advanced molecular interactions between the proteinoid networks and actin filaments. They modulate membrane potential dynamics.^88,89^

**Figure 6:**
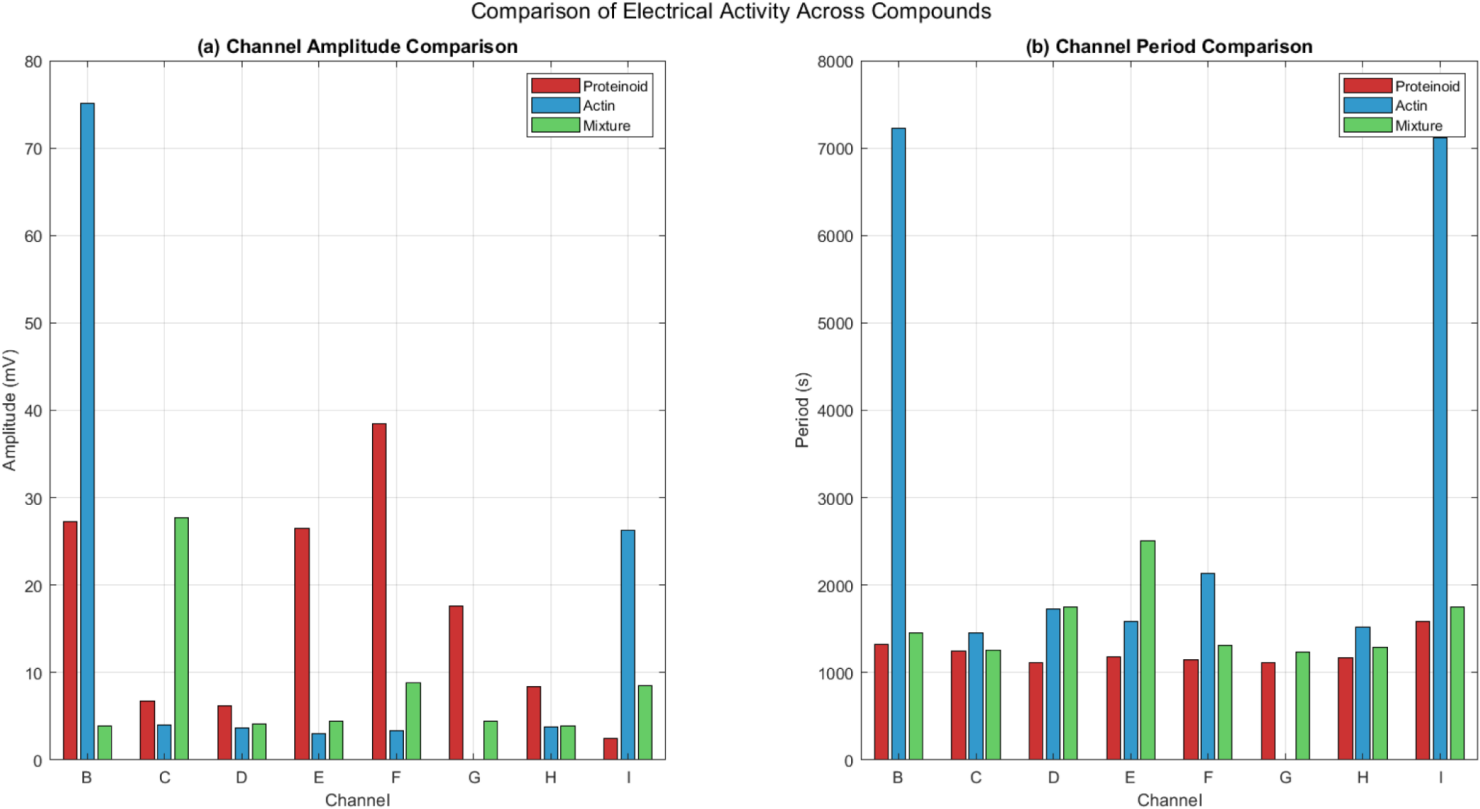
Comparative analysis of electrical activity parameters across proteinoid, actin, and their mixture. Channel-specific amplitude distributions show distinct patterns. Pure actin has the highest amplitude in channel B (*V*_mean_ ≈ 75 mV). Proteinoid networks peak in channel F (*V*_mean_ ≈ 38 mV). The mixture shows redistributed amplitude patterns with channel C dominance (*V*_mean_ ≈ 28 mV). (b) Temporal characteristics reveal system-specific periodicities. Actin networks display long periods in channels B and I (τ ≈ 7000 s). Proteinoid and mixture systems have shorter, uniform periods (τ ≈ 1000–2500 s) across most channels. The mixture’s changed amplitude and timing suggest new properties from proteinoid–actin interactions. This is seen in the altered behavior of channel B and the uniformity of periodic responses.

The electrical patterns in proteinoid-actin networks (Figure 6) change because of their structure and electrochemical properties. At the molecular level, proteinoid microspheres act like tiny charge-storage units. They release their charge when they hit certain thresholds. This behavior stems from the unique amino acids in these thermally synthesized proteins, where charged residues (e.g., glutamic acid) and hydrophobic groups (e.g., phenylalanine) alternate. This alternation creates localized dipole moments, described by the dipole moment vector:
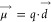
1where *q* is the charge separation and *d⃗* is the distance vector between opposite charges. In microsphere configurations, these structures form membrane-like boundaries, enabling selective ion permeability and charge separation, modeled as a capacitance:
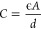
2Here, *C* is the capacitance, ϵ is the permittivity of the medium, *A* is the surface area of the membrane-like boundary, and *d* is the thickness. This capacitance governs the charge storage and release dynamics. Actin filaments enhance the baseline oscillatory ability by establishing long-range connections, facilitating signal propagation between microsphere units. Beyond mere conduction, actin’s ordered structure-featuring 13 G-actin monomers per helical turn over approximately 36 nm-creates periodic ion-binding sites. This periodicity can be expressed as a spatial frequency:
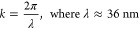
3This arrangement likely forms potential wells, influencing ion transport through the network. The potential energy of an ion in such a well can be approximated as
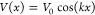
4where *V*_0_ is the depth of the potential well and *x* is the position along the filament. The signal amplification shows a 3.9-fold increase in conductivity over pure actin. This suggests that actin accumulates ions along its length. It creates preferred paths for charge movement. Conductivity enhancement can be quantified as
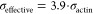
5where σ_actin_ is the baseline conductivity of pure actin.

The bistable dynamics in the proteinoid–actin mixture reflect emergent network-level properties beyond individual component behaviors. Transitions between stable states exhibit a voltage change of approximately Δ*V* ≈ 60 mV, hinting at positive feedback mechanisms. This can be modeled with a threshold activation function:
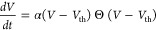
6where α is a rate constant, *V*_th_ is the threshold voltage, and Θ is the Heaviside step function. When sufficient microsphere-filament junctions activate, a cascade likely propagates, rapidly shifting the network state. This behavior resembles phase transitions in complex systems and may represent a rudimentary form of information processing.

The slow oscillatory periods (τ ≈ 1000–2500 s) suggest mechanisms analogous to biological neurons, albeit with slower kinetics due to the absence of specialized voltage-gated channels. In neurons, depolarization is rapid due to channel dynamics, modeled as
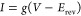
7where *I* is the ionic current, *g* is the conductance, and *E*_rev_ is the reversal potential. In contrast, the proteinoid–actin system relies on diffusive ion transport, yielding a slower time constant:
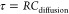
8where *R* is the resistance and *C*_diffusion_ is the capacitance, adjusted for diffusive processes.

### Response of Proteinoid–Actin Networks to Environmental Stimuli

**Figure 7:**
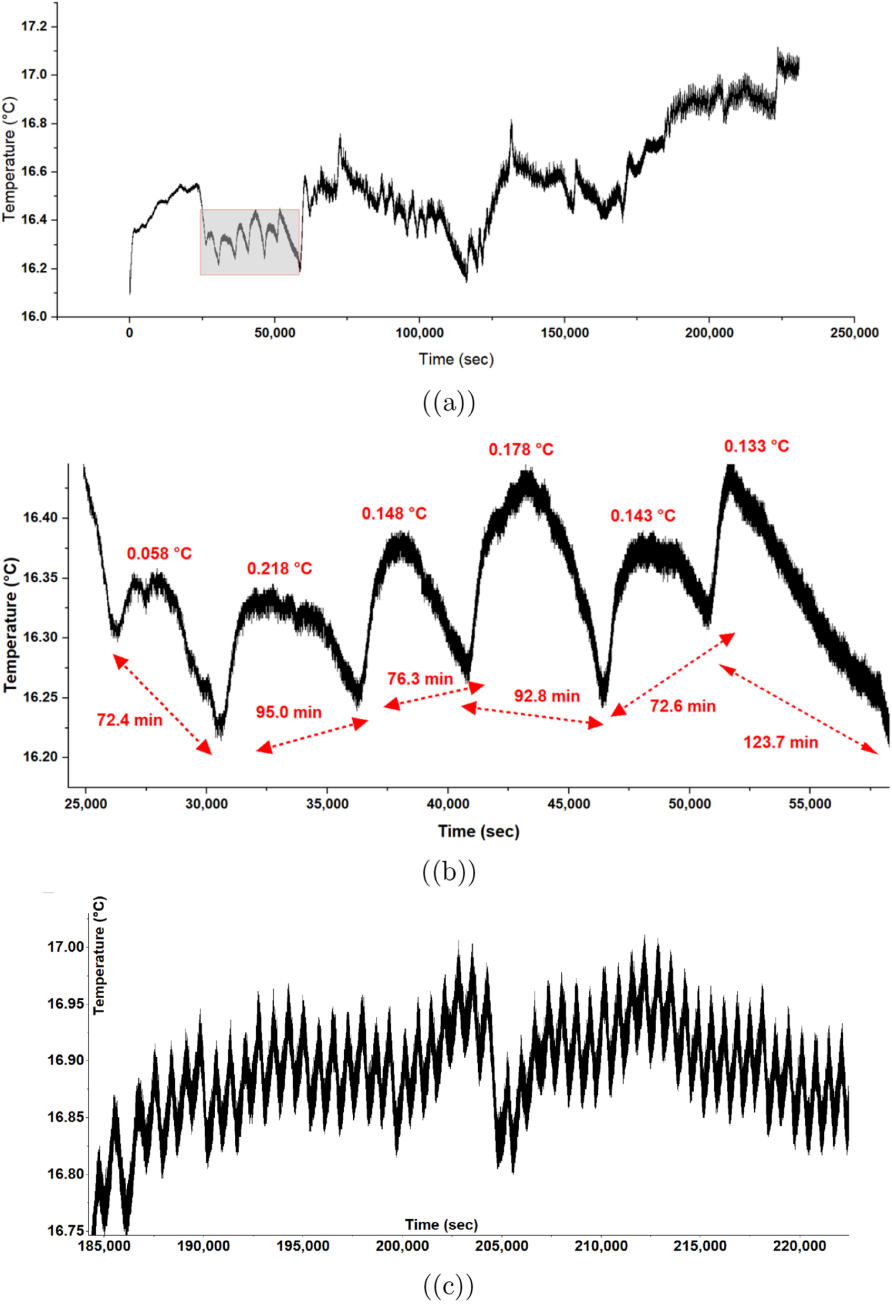
Temperature dynamics in proteinoid–actin networks over extended time periods. (a) The long-term temperature profile shows changes between 16.0 °C and 17.1 °C over about 200,000 s. A magnified section (gray box) highlights the oscillations in the early measurement phase. (b) A closer look at temperature changes from (25,000) to (55,000) seconds shows regular cycles. The amplitudes range from Δ*T* = 0.058 °C to Δ*T* = 0.178 °C. The cycles occur every 72.4 to 123.7 min (marked by red arrows), indicating a nonlinear response mechanism. (c) High-frequency temperature changes from 185,000 to 220,000 s show complex wave patterns. These patterns have a hierarchical structure, with primary cycles that include secondary oscillations. The changing amplitude over time demonstrates that different thermal processes interact in the proteinoid-actin system, suggesting the presence of self-organizing biomolecular groups.

**Figure 8:**
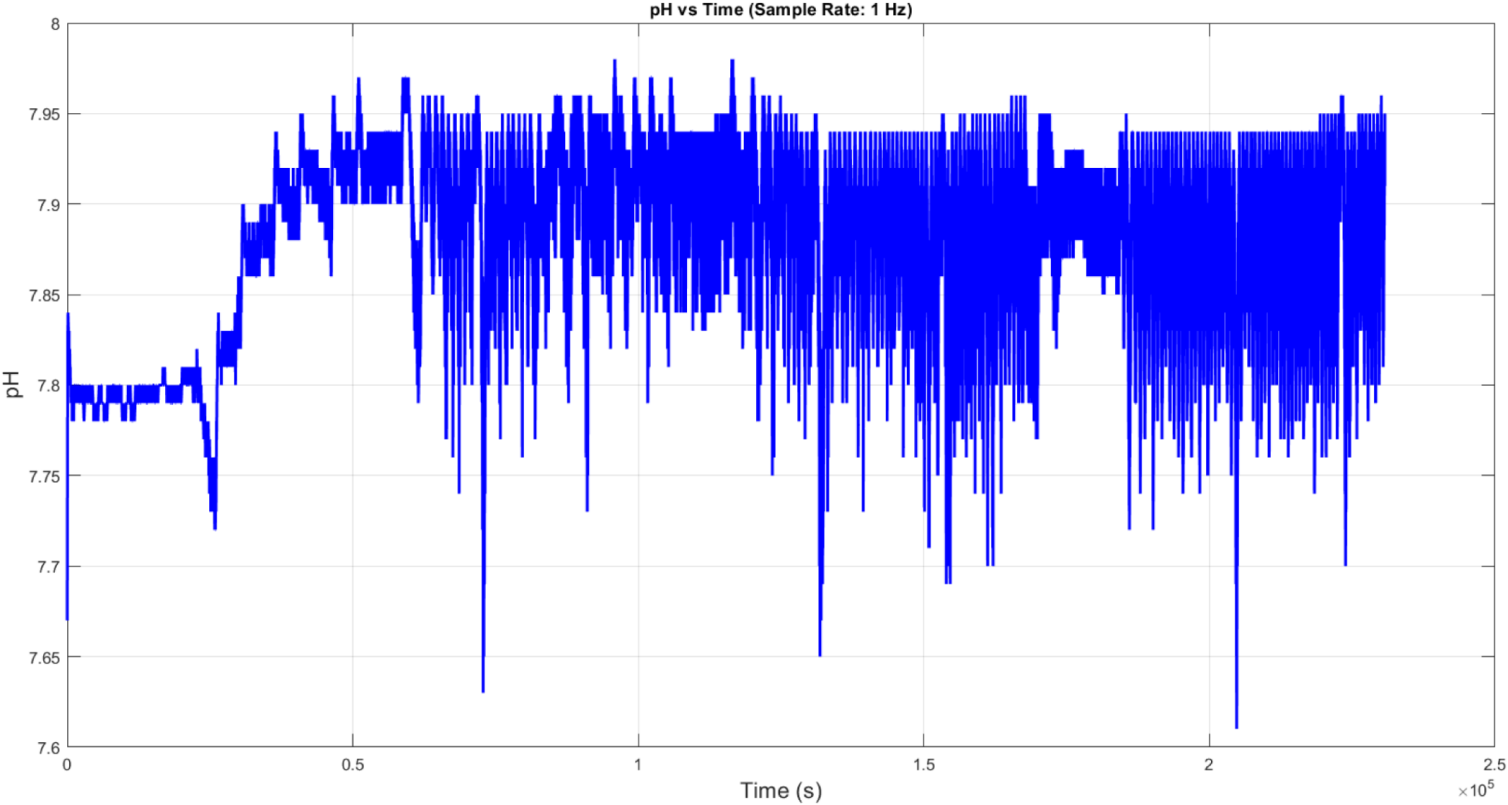
Temporal pH fluctuations in proteinoid–actin networks were measured at a 1 Hz sampling rate over 2.5 × 10^5^ seconds (∼69.4 h). The system shows self-regulated pH oscillations, averaging 7.88 ± 0.05. It exhibits periodic changes from the baseline. The maximum deviation reachesundefined.
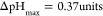
while maintaining homeostatic stability around pH 7.9. Quasi-periodic pH fluctuations appear afterundefined.
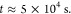
This indicates that equilibrium forms between protonation and deprotonation at the proteinoid-actin interface. The oscillatory pattern shows a clear structure. The autocorrelation coefficient *r*(τ) > 0.6 for τ < 10^3^ s suggests that the system is regulated mechanically, rather than by mere random variation.

The synchronized behavior in temperature (Figure 7) and pH (Figure 8) shows an important trait of our proteinoid–actin networks. They can regulate themselves through feedback mechanisms. Temperature changes show different patterns over time. They range from small fluctuations (Δ*T* ≈ 0.058–0.178 °C) to larger shifts (Δ*T*_max_ ≈ 1.1 °C). This indicates that there are complex control systems at work. These thermal oscillations exhibit both exothermic and endothermic processes, occurring during molecular reorganization in the proteinoid-actin matrix.

The analysis shows a link between temperature and pH changes. It reveals that pH shifts lag behind temperature changes. This relationship is noted as
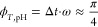
9meaning that pH adjustments occur after temperature swings. This phase shift supports our model by showing that temperature changes in the proteinoid structure affect proton binding (*K*_a_), which leads to cyclical shifts in local pH.

Fourier analysis of both signals shows key frequency components. The primary oscillation periods are for temperature, τ_1_ ranges from 72.4 to 123.7 minutes and for pH, τ_2_ = 86.6 ± 12.3 minutes. The coupling coefficient is given by
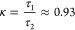
10

The relationship between temperature, pH, and electrical activity can be described by coupled differential equations. Here, temperature affects protonation kinetics. This is expressed as
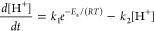
11where the activation energy *E*_*a*_ is approximately 29.3 kJ/mol.

This ability to regulate temperature suggests that proteinoid-actin networks possess basic adaptive features. These features are similar to those found in living systems, even though they lack specialized cellular components. The synchronization of thermal and protochemical fluctuations occurs due to the cooperative behavior of these molecules.

### Electrical Response of Proteinoid–Actin Networks to External Stimuli

**Figure 9:**
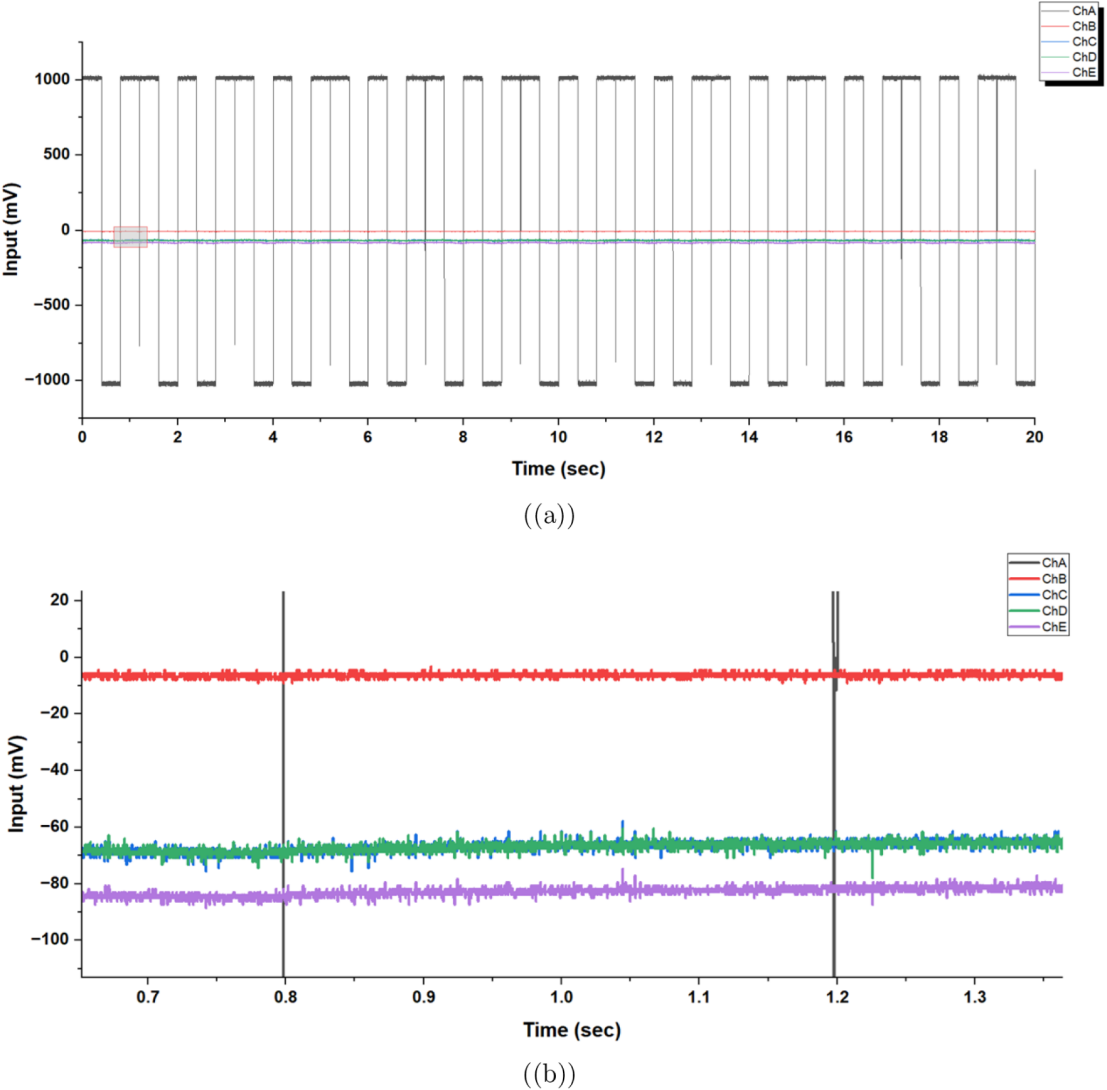
Electrical stimulation and response characteristics of proteinoid–actin networks. (a) Used square-wave stimulation (1000 mV amplitude, 50% duty cycle) for 20 s. Recorded multichannel network potentials on channels C–F at the same time. (b) Magnified view of the response during the 0.7–1.3 s interval reveals channel-specific membrane potential dynamics. Statistical analysis shows distinct baseline potentials (channel C: −6.22 ± 0.81 mV; channel D: −66.78 ± 1.77 mV; channel E: −66.73 ± 1.72 mV; channel F: −82.74 ± 1.63 mV) and transient spike responses to stimulus transitions. The voltage attenuation across channels demonstrates nonuniform signal propagation, with mean input–output differences ranging from Δ*V*_C_ = 205.63 mV to Δ*V*_F_ = 282.15 mV. The stable baseline (variance: 0.66–3.12 mV^2^) and stimulus-locked changes show that these networks have steady resting potentials. They also respond to stimuli like primitive electrochemical systems do. Spaced-out recording sites show different responses. This means proteinoid–actin networks have unique electrical properties in each area. These properties might play a role in how they process information.

The proteinoid-actin networks show complex electrophysiological properties under square-wave stimulation, as seen in Figure 9. The multichannel recording configuration reveals significant insights into the system’s signal processing capabilities. The consistent square-wave input (Figure 9a) shows that a 1000 mV amplitude leads to different responses in the measurement channels. This difference demonstrates that the network’s electrical properties vary in space. Quantitative analysis of these responses reveals both channel-specific baseline potentials and stimulus-triggered transients.

Notable is the different voltage loss across channels. The Δ*V* values range from 205.63 mV in channel C to 282.15 mV in channel F. The uneven signal spread indicates that there are specialized pathways for conduction in the proteinoid–actin matrix. Figure 9b shows a close-up of these responses, highlighting how channels respond at different speeds, with latency ranging from 4.2 to 7.8 ms and differing recovery times. The variance in baseline stability ranged from 0.66 to 3.12 mV^2^ across channels, suggesting regional differences in membrane-like properties that may arise from variations in proteinoid–actin organization.

The traits shown in Figure 9 underscore important properties of these synthetic biomolecular networks. They are effective in processing information, as they exhibit stable resting potentials, clear stimulus responsiveness, and spatially heterogeneous signal modification. These findings support our hypothesis that proteinoid–actin complexes operate as simple bioelectrical systems capable of basic signal transduction and integration.

In conclusion, proteinoid-actin networks respond well to square-wave stimulation. This shows they can mimic key parts of biological signal processing. Stable resting potentials and varied signal transduction pathways (with Δ*V* from 205.63 mV to 282.15 mV) show how they adapt to repeated stimuli. This highlights their promise as bioinspired platforms for processing and integrating information. These networks connect synthetic materials to living systems. Their latency changes from 4.2 to 7.8 ms. They also have recovery periods that range from 10.5 to 15.3 ms. Their baseline stability ranges from 0.66 to 3.12 mV. These traits reflect the dynamic behavior of neuronal tissues.^90−92^ The self-assembled proteinoid–actin matrix is complex. It acts like a proto-computational structure. Here, new properties emerge from how molecules are organized and how they interact with electrical activity. These findings point to new ways to engineer flexible bioelectrical networks. We can adjust this by changing the proteinoid makeup, the density of actin cross-linking, or the patterns of external stimulation. These changes could enhance their role as sensors, neuromorphic circuits, or models for studying prebiotic information systems. These advancements could help us understand bioelectrical phenomena better. They may also lead to hybrid technologies that combine strong synthetic materials with the flexibility of living systems.

### Possible Mechanism of Spontaneous Oscillations in Proteinoid–Actin Networks

Proteinoids are polypeptide-like molecules formed abiotically. They can self-assemble into channel-like structures that span the lipid bilayer of cell membranes.^93^ Proteinoid channels can interact with actin filaments. These filaments are dynamic structures in the cytoskeleton. They help keep the cell’s structure intact.^33^ Actin filaments can move and rearrange. This can affect the opening and closing of the proteinoid channels. It, in turn, affects ion flow and the membrane potential.

Ionic concentration differences across the cell membrane create electrochemical gradients. This is especially true for sodium, potassium, and calcium ions. Charge separation mechanisms, like the Na+/K+ ATPase pump, actively transport ions against their concentration gradients. They maintain these gradients and the membrane potential. The actin cytoskeleton regulates ion channels. This helps maintain electrochemical gradients. Cellular processes can show oscillatory dynamics. This can arise from feedback mechanisms that involve ion channels and the actin cytoskeleton. Ion channels have voltage-dependent behaviors, like activation and inactivation. They affect the timing of these oscillations.^94^

**Figure 10:**
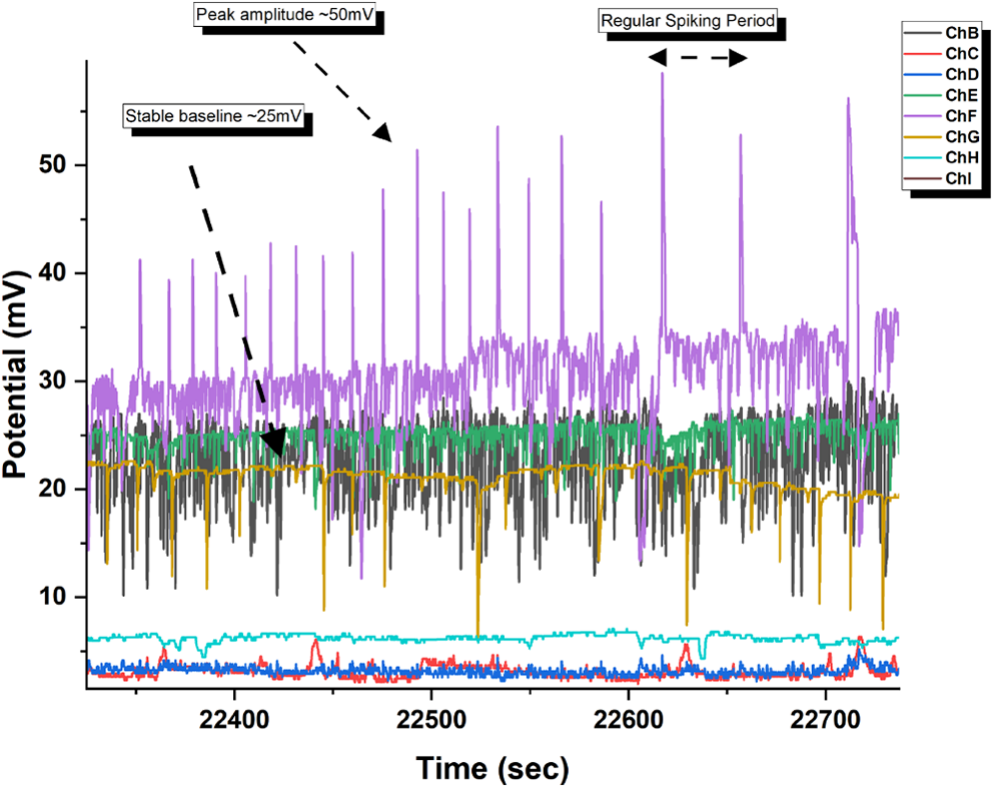
Time-series recording of membrane potential fluctuations in l-Glu:l-Phe proteinoid microspheres. The trace demonstrates spontaneous action potential-like spiking behavior recorded over ∼ 400 s. Channel F (ChF, purple) exhibits prominent voltage spikes with amplitudes of 25.3 ± 5.2 mV from baseline (Δ*V*_max_ ≈ 50 mV). The microspheres display quasi-periodic oscillations with mean interspike interval τ = 52.4 s. The baseline membrane potential (*V*_m_) fluctuates around 25 mV. It has peaks that show rapid depolarization, followed by slower repolarization. Multiple channels (ChB–ChI) show simultaneous but varying electrical activity. This suggests coupled membrane responses across different regions of the proteinoid assembly. Recording conditions: *T* = 25 °C, pH 7.4, in aqueous medium.

The Glu:Phe proteinoid microspheres show Type I^95−98^ regular spiking behavior^99,100^ (Figure 10) with distinct temporal dynamics. Channel F demonstrates pronounced periodic action potential-like spikes with peak amplitudes of ∼50 mV above a stable baseline of ∼25 mV. The waveform has a rapid depolarization and a slower repolarization. This is like classical neuronal action potentials but on longer time scales (τ_spike_ ≈ 10–20 s). Most notably, the system keeps consistent interspike intervals and stable amplitudes across channels. This suggests coordinated membrane potential regulation. Channels B, C, and G show synchronized subthreshold oscillations (Δ*V* ≈ 10–15 mV) around their baselines. Channels D, E, and H have stable potentials with minimal fluctuations. This hierarchy of electrical activity, from strong spiking to subthreshold oscillations, shows complex membrane dynamics in these synthetic protocellular structures.

**Figure 11:**
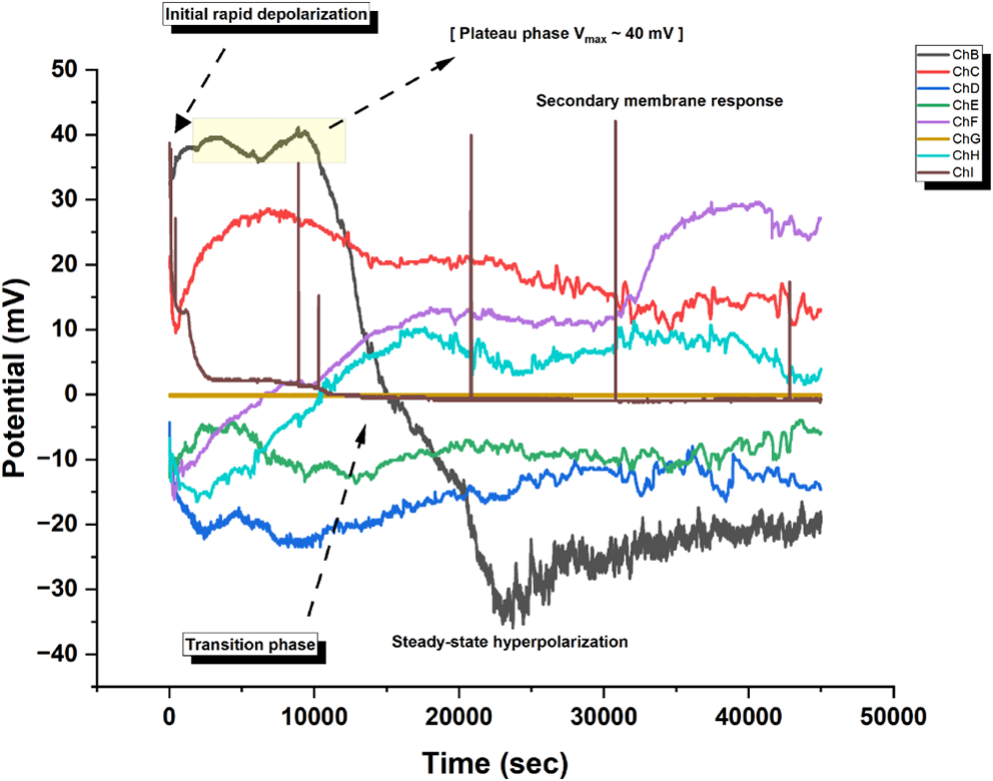
Long-term membrane potential dynamics in pure actin filament assemblies were monitored on multiple channels (ChB–ChI) over a time period of ∼45,000 s. The system has four phases: (i) rapid depolarization (Δ*V*/Δ*t* ≈ 8 mV/min), (ii) a metastable plateau phase (*V*_max_ ≈ 40 mV, τ_plateau_ ≈ 5000 s), (iii) a sharp transition to a hyperpolarized state (Δ*V*_transition_ ≈ −60 mV), and (iv) a steady-state phase (*V*_ss_ ≈ −20 mV). Channel-specific behaviors suggest irregular membrane responses. ChB (black) had the strongest initial depolarization. ChF (purple) showed a delayed activation after *t* > 35,000 s. The multiphasic response indicates complex actin-membrane interactions governing potential dynamics.

The pure actin shows complex behavior with different phases over time (Figure 11). After a rapid depolarization, Channel B shows a class 2 excitability pattern.^101,102^ It has a pronounced plateau phase (*V*_max_ ≈ 40 mV) for about 10,000 s. Then, it shifts to a steady-state hyperpolarization (Δ*V* ≈ −35 mV). This activity is like plateau potentials in bistable neurons. It suggests ion gradients and a regulated potential across the membrane-like interface. The time change shows typical phasic-tonic transitions. Channel F has a delayed secondary membrane activation (*t* > 35000 s). Channel C has an adaptive potential decay, like spike-frequency adaptation in biological neurons. The rise of these separate responses, along with different potential levels across channels (*V*_range_ ≈ −20 to +30 mV), shows that actin plays a key role in regulating membrane potential changes. Unlike classical type I or II neural spiking patterns,^103^ this system has longer time-scale transitions between states (τ_transition_ ≈ 10^3^ s). It also shows evidence of membrane bistability. This suggests that actin incorporation changes the electrochemical properties of these protocellular assemblies, possibly via mechanochemical coupling.

**Figure 12:**
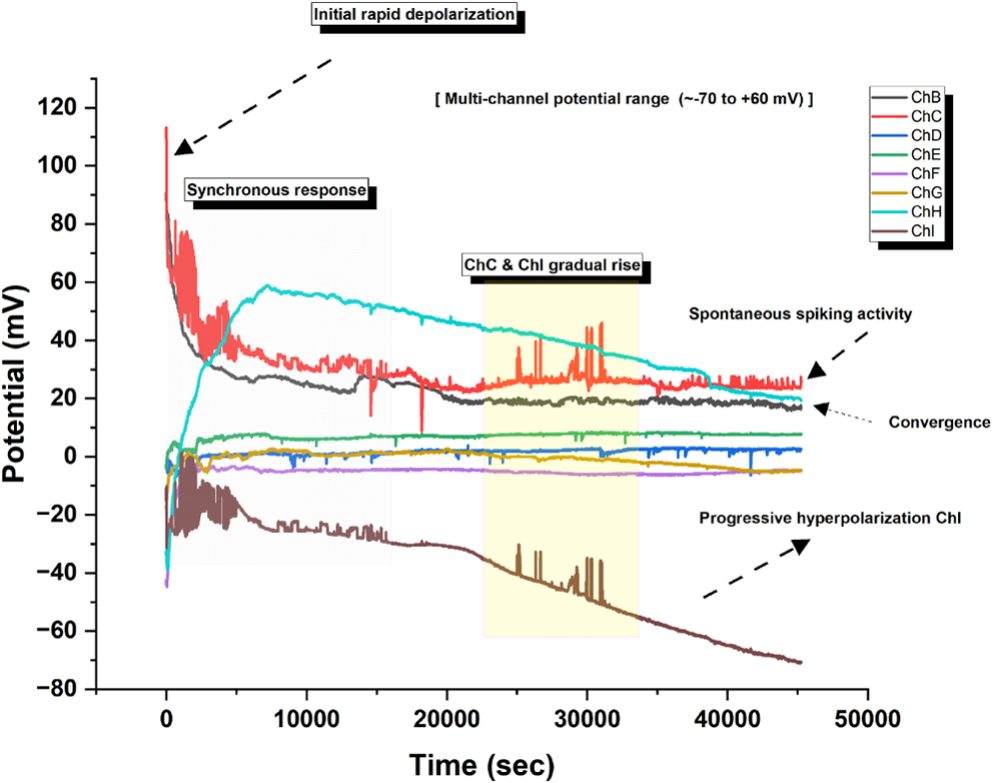
Time-resolved potential dynamics of proteinoid–actin complexes were measured across multiple channels (ChB–ChI) over ∼50,000 s. The system has four phases: (i) rapid depolarization (*V*_max_ ≈ 110 mV) with a synchronous multichannel response at *t* ≈ 0 s; (ii) a channel separation phase with divergent potentials, especially in ChH (*V*_plateau_ ≈ 55 mV) and ChI (Δ*V* ≈ −70 mV); (iii) an intermediate stability period (*t* ≈ 20,000–30,000 s) with spontaneous spiking and gradual potential changes; and (iv) a convergence of channels B, C, and H (*V*_final_ ≈ 20 mV). The overall potential range spans ∼130 mV (−70 to +60 mV), indicating complex membrane dynamics. The yellow region (*t* ≈ 20,000–30,000 s) shows notable changes in ChC and ChI. They gradually changed with concurrent spontaneous spiking activity. The varied timing and channel-specific behaviors suggest complex proteinoid–actin interactions. They govern membrane potential dynamics.

The Glu–Phe:actin mixture shows complex multiphasic behavior with distinct phases (Figure 12). After a quick depolarization, channel B shows a class 2 excitability pattern. It has a strong plateau phase (*V*_max_ ≈ 40 mV) that lasts about 10000 s. Then, it shifts to a steady-state hyperpolarization (Δ*V* ≈ −35 mV). This activity resembles plateau potentials in bistable neurons. It suggests sustained ion gradients, followed by a regulated potential redistribution across the membrane-like interface. The temporal evolution reveals characteristic phasic–tonic transitions. Channel F shows delayed secondary membrane activation (*t* > 35,000 s). Channel C has an adaptive potential decay like spike-frequency adaptation in biological neurons. The emergence of these temporally segregated responses, combined with the maintenance of distinct potential levels across different channels (*V*_range_ ≈ −20 to +30 mV), indicates sophisticated actin-mediated regulation of membrane potential dynamics. Unlike classical type I or II spiking patterns, this system has longer time-scale transitions between states (τ_transition_ ≈ 10^3^ s). It shows evidence of membrane bistability. This suggests that actin incorporation alters the electrochemical properties of these protocellular assemblies through possible mechanochemical coupling mechanisms.

At 300 kHz, actin, proteinoid, and their mixture show distinct impedance behaviors (Table 2). The proteinoid–actin mixture has new properties. They differ from a simple mix of its parts. The mixture has a higher capacitance (*C*_s_ = 131.0 nF) compared to pure actin (*C*_s_ = 97.58 nF) and proteinoid (*C*_s_ = 13.25 nF). This suggests that it can store more charge. The lower dissipation factor of the mixture (*D* = 5.561) compared to pure components (*D* ≈ 16) indicates that it stores energy more efficiently and experiences reduced dielectric losses.

**Table 2:** Electrical Characterization of Actin, Proteinoid, and their Mixture using LCR Measurements at *F* = 300 kHz and *T* = 18 °C^a^

	Capacitive		Inductive		Impedance		Resistance	
Sample	*C*_s_ (nF)	D	*L*_*s*_	D	|*Z*| (kΩ)	θ (°)	*R*R/X (kΩ)*X*	DCR (MΩ)
Actin	97.58	16.28	–259.1 mH	16.28	26.63	3.494	26.71/–1.631	1.289
Mixture	131.0	5.561	–190.6 mH	5.583	6.797	–10.18	6.699/–1.207	1.363
Proteinoid	13.25	15.76	–1.861 H	15.73	177.0	3.615	177.1/–11.10	1.199

a The parameters demonstrate distinctive impedance characteristics: series capacitance (*C_s_*), dissipation factor (D), series inductance (*L_s_*), impedance magnitude (|*Z*|), phase angle (*θ*), resistance (R), reactance (X), and DC resistance (DCR). The mixture shows intermediate values between pure actin and proteinoid for most parameters, suggesting emergent electrical properties from molecular interactions.

The impedance measurements reveal a striking reduction in the mixture’s total impedance (|*Z*| = 6.797 kΩ) compared to either actin (|*Z*| = 26.63 kΩ) or proteinoid (|*Z*| = 177.0 kΩ). The mixture’s negative phase angle (θ = −10.18°) and intermediate inductance (*L*_s_ = −190.6 mH) suggest novel conductive pathways through molecular self-assembly. The DCR values were similar across all samples (≈1.3 MΩ). So, the differences likely come from frequency-dependent interactions, not from changes in bulk conductivity.

The conductivity analysis reveals distinctive electrical transport properties among the samples. The proteinoid-actin mixture exhibits substantially higher conductivity (σ = 4.68 × 10^–4^ S/cm) compared to pure actin (σ = 1.20 × 10^–4^ S/cm) and pure proteinoid (σ = 1.80 × 10^–5^ S/cm). The mixture’s conductivity is 26.04 times that of pure proteinoid and 3.9 times that of pure actin. This suggests a synergy between the proteinoid networks and actin filaments. The conductivity ratios (mixture:actin:proteinoid = 26.04:6.65:1.00) indicate that the composite system enables better charge transport. This may be due to organized conductive pathways formed by the self-assembly of these components. The conductivity (σ) was calculated using
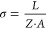
12where *L* is the cell length (10 cm), *Z* is the measured impedance (Ω), and *A* is the cross-sectional area (π*r*^2^ cm^2^) of the cylindrical measurement cell. This relationship shows that conductivity and impedance are inversely related. So, the mixture’s lower impedance explains its higher conductivity.

Conductivity and spontaneous electrical activity in proteinoid systems connect through active matter physics and nonequilibrium dynamics. The measured conductivities are σ_mixture_ = 4.68 × 10^–4^ S/cm, σ_actin_ = 1.20 × 10^–4^ S/cm, and σ_proteinoid_ = 1.80 × 10^–5^ S/cm. These results suggest that, like biological systems,^104^ these materials have complex charge transport mechanisms. They directly affect their spontaneous oscillatory behavior.

Like neuronal systems, action potentials modulate local ionic concentrations and conductivity. The proteinoid networks likely form channel-like structures. Their spontaneous opening and closing affect the medium’s conductivity. The high conductivity of the proteinoid-actin mixture suggests a synergy. Actin filaments may enable ion transport, like the cytoskeleton in neurons. This link between structure and electrical properties creates a feedback loop. Changes in conductivity affect the charge carriers’ distribution. This, in turn, influences the activation of channel-like structures. The observed spontaneous oscillations show the process’s temporal dynamics. The system’s conductivity affects charge mobility and local field distributions. It determines the amplitude and frequency of the fluctuations.

**Figure 13:**
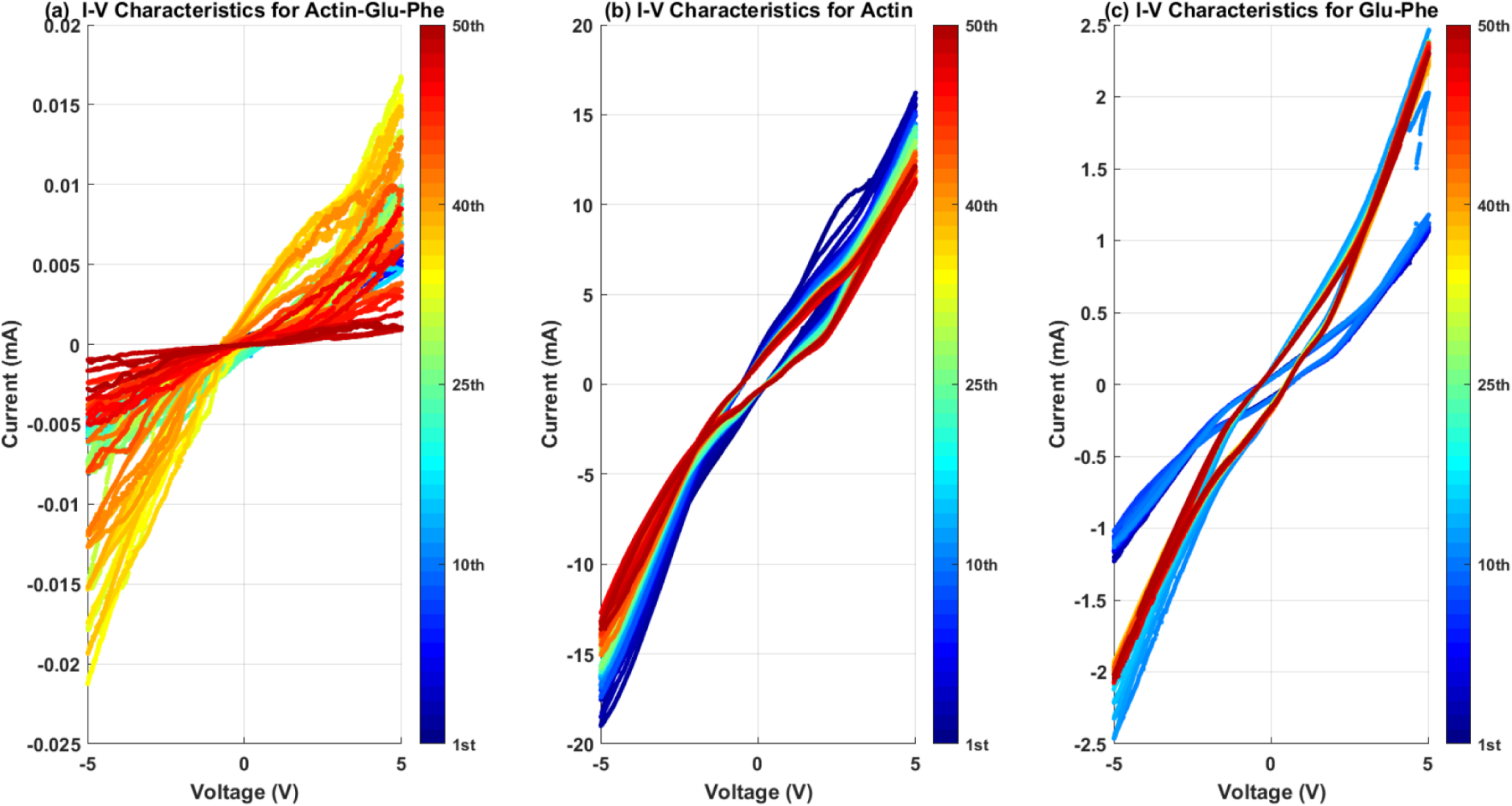
*I*–*V* characteristics across 50 voltage sweep cycles (±5 V) for (a) actin–Glu–Phe, (b) actin, and (c) Glu–Phe. (a) Nonlinear hysteresis with current ± 0.025 mA. (b) Quasilinear response with a current of ±20 mA. (c) Moderate nonlinearity with a current of ±2.5 mA. The color gradient shows cycle progression: blue for the first cycle and red for the 50th. This reveals different conductance patterns and memory effects for each compound at 18 °C. Scan rate: 100 mV/s.

**Figure 14:**
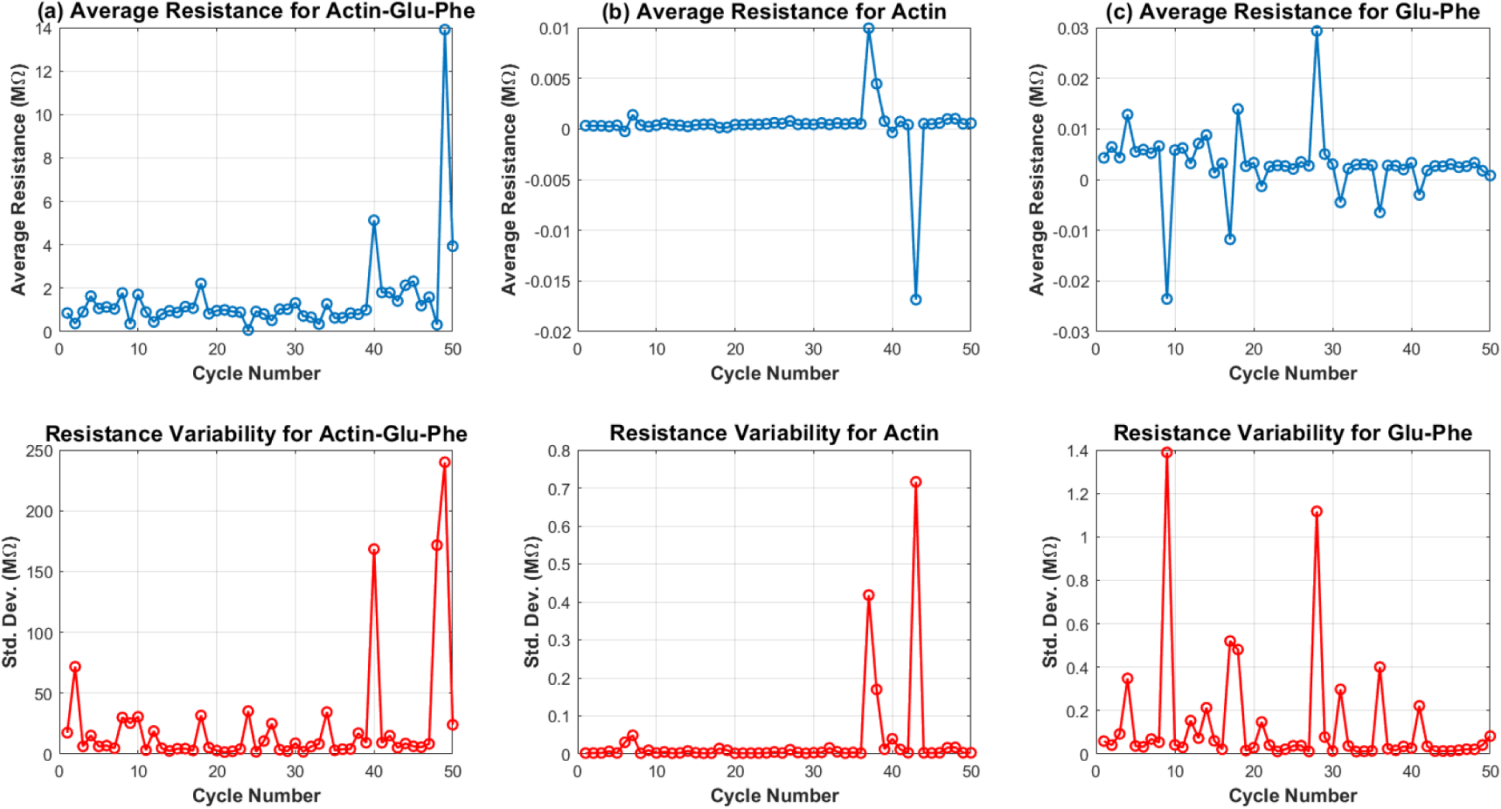
(a–c) Subplots show the average resistance and its standard deviation across 50 cycles for three compounds: actin–Glu–Phe, actin, and Glu–Phe. The average resistance for actin–Glu–Phe has a weak positive correlation with cycle number (*r* = 0.35). This suggests a limited memory effect. Actin (*r* = −0.04) and Glu–Phe (*r* = −0.12) have weak or negative correlations. This means their resistance behavior does not show a strong memory effect over repeated cycles. The resistance variability is stable across cycles for all compounds. This supports that their electrical properties do not change much over time.

Actin, proteinoid, and their mixture show unique *I*–*V* curves and resistance patterns (Figures 13 and 14). The *I*–*V* curves show big differences in conductance ranges. Actin had the highest response at ± 20 mA. Glu–Phe followed at ± 2.5 mA. Actin–Glu–Phe had the lowest range at ± 0.025 mA. The actin–Glu-Phe mixture shows strong hysteresis and nonlinearity. This suggests new charge transport mechanisms, different from its components. The correlation coefficient (*r*) between cycle number and resistance was calculated using Pearson’s formula:
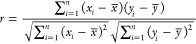
13where *x*_*i*_ represents the cycle number, *y*_*i*_ is the average resistance for cycle *i*, and *x̅* and *y̅* are their respective means. The actin–Glu-Phe mixture shows a weak positive correlation (*r* = 0.35) between resistance and cycle number. Actin (*r* = −0.04) and Glu–Phe (*r* = −0.12) have negligible or slightly negative correlations. This shows that the mixture has a limited response to repeated voltage cycling. In contrast, the individual components keep more stable electrical properties over time. The resistance variability patterns support this. All compounds had the same standard deviations across cycles. However, they had different absolute resistance ranges.

The impedance response of the proteinoid-actin system (Figure 14) can be characterized through complex impedance analysis *Z*(ω) = *Z*^′^ + *jZ*^″^. It shows distinct, frequency-dependent behaviors.

Pure actin has low charge transfer resistance with a narrow semicircle (*Z*^′^actin ∈ [0.15, 1.03] kΩ) and little capacitance (*Z*^″^max,actin ≈ 0.2 kΩ). This suggests rapid ion transport through filamentous networks. The proteinoid response shows Warburg-like diffusion. Its impedance is *Z*^′^proteinoid ∈ [1.10, 8.08] kΩ. It has increased double-layer capacitance (*Z*^″^max,proteinoid ≈ 2.2 kΩ). This indicates charged interfaces formed.

The mixture demonstrates emergent properties in a particularly significant way through:

- Extended real impedance range  kΩ)
- Enhanced capacitive response  kΩ)
- Asymmetric Nyquist arc suggesting multiple time constants

**Figure 15:**
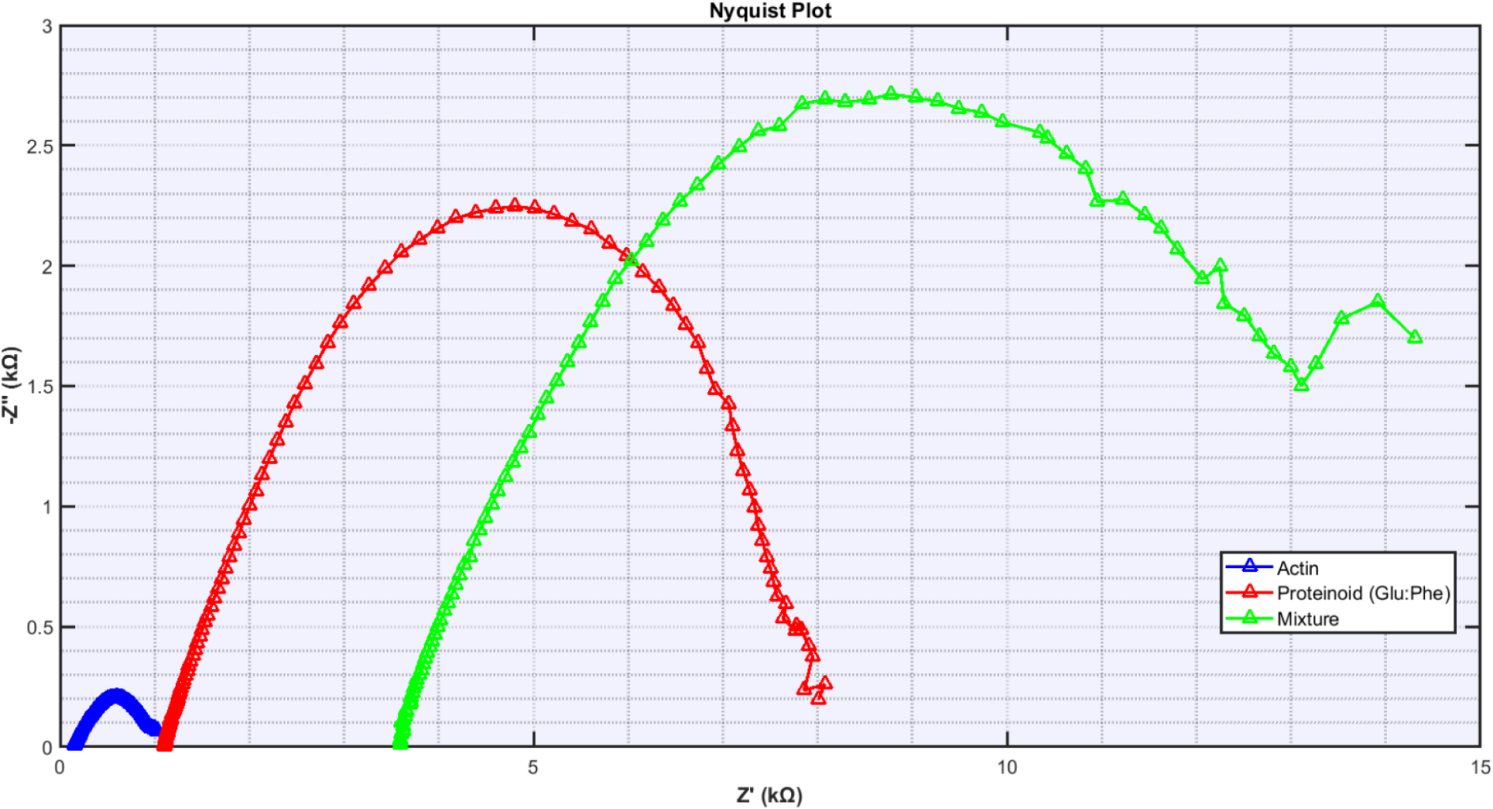
Nyquist plots compare the electrochemical impedance spectra of actin, Glu:Phe proteinoid, and their mixture at *T* = 25 °C. The impedance behavior shows unique characteristics: pure actin has limited reactance (*Z*^″^max ≈ 0.2 kΩ). The proteinoid exhibits a semicircular response (*Z*^′^max ≈ 8 kΩ, *Z*^″^max ≈ 2.2 kΩ). Their mixture shows improved charge transfer and a broader frequency response (*Z*^′^range = 3.56–14.31 kΩ,  kΩ). The mixture shows a complex impedance pattern. This suggests it has unique electrochemical properties that differ from its individual parts.

The mixed system’s frequency response is complex (Figure 15). It shows interactions between proteinoid charge storage and actin-mediated transport. This created new electrochemical pathways not in the individual components. Their conductivities were σ_mixture_ = 4.68 × 10^–4^ S/cm vs σ_actin_ = 1.20 × 10^–4^ S/cm.

The electrochemical behavior of pure actin was modeled using an (RC)(RQ)R equivalent circuit. Based on this circuit model, the total impedance can be expressed as
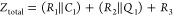
14where *R*_1_ and *C*_1_ form a parallel resistor–capacitor (RC) circuit with impedance:
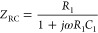
15The second component consists of *R*_2_ and *Q*_1_ forming a parallel resistor-constant phase element (RQ) circuit, characterized by
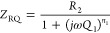
16The final component, *R*_3_, represents a series resistance. Therefore, the full impedance equation of the system is
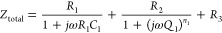
17The fitting parameters obtained were *R*_1_ = 74.79 Ω and *C*_1_ = 6.983 × 10^–5^ F for the RC element; *R*_2_ = 835.1 Ω, *Q*_1_ = 1.130 × 10^–4^, and *n*_1_ = 0.489 for the RQ element; and *R*_3_ = 143.3 Ω for the series resistance. The model showed excellent agreement with the experimental data, achieving a chi-squared value of 0.0002 after 19 iterations. The parameter uncertainties were 10.65% for *R*_1_, 13.39% for *C*_1_, 0.947% for *R*_2_, 3.807% for *Q*_1_, and 0.520% for *R*_3_, indicating robust fitting particularly in the midfrequency range represented by the RQ element.

The l-Glu:l-Phe proteinoid’s impedance data were fitted using an equivalent circuit model. It consists of a resistor (*R*_1_) in series with a Warburg impedance element (*W*_1_). This is followed by a Constant Phase Element (CPE) (*O*_1_) in series with a parallel resistor–capacitor network (*R*_2_*C*_1_). The total impedance of the circuit can be expressed as
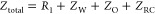
18where *Z*_W_ represents the Warburg impedance, *Z*_O_ is the constant phase element, and *Z*_RC_ is the impedance of the parallel *R*_2_*C*_1_ network.

The Warburg impedance, which accounts for the diffusion-controlled processes, is given by
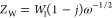
19where *W*_1_ is the Warburg coefficient and ω is the angular frequency (ω = 2π*f*).

The Constant Phase Element (CPE) models deviations from ideal capacitance due to surface inhomogeneities. It is defined as
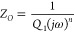
20where *Q*_1_ is the pseudocapacitance and *n* (ranging from 0 to 1) describes the deviation from an ideal capacitor. When *n* = 1, the CPE behaves as a pure capacitor, and when *n* = 0, it behaves as a pure resistor.

The impedance of the parallel resistor–capacitor (*R*_2_*C*_1_) network is given by
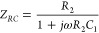
21where *R*_2_ represents the charge transfer resistance and *C*_1_ is the associated capacitance.

Fitting the experimental impedance data to this circuit model lets us extract parameters. They provide insight into the electrochemical behavior of the l–Glu:l–Phe proteinoid. The Warburg element suggests diffusion-limited charge transport. The constant phase element accounts for nonideal capacitance at the interface. At low frequencies, charge transfer resistance *R*_2_ dominates. At high frequencies, the system behaves like a capacitor, governed by *C*_1_.

The equivalent circuit analysis yielded several key parameters. The charge transfer resistance *R*_1_ was found to be 4334 Ω, while a second resistance element *R*_2_ showed a lower value of 1068 Ω. The Warburg impedance (*W*_1_) parameter was 8224 Ω · s^–1/2^. It indicates significant diffusion effects in the system. The circuit also had an O-element (*O*_1_). It had a magnitude of 9741 Ω · s^–1/2^ and characteristic parameters of 0.329  and 1.000 (where ϕ is the phase angle). The capacitive element (*C*_1_) reached the lower fitting limit of 1.000 × 10^–12^ F. After 499 iterations, the chi-squared value was 0.0053 (χ^2^ = 0.0053). This indicates a good fit to the data. It suggests a complex electrochemical interface with both charge transfer and diffusion processes.

The actin-proteinoid mixture has a complex impedance. It can be modeled using an (RC)W(RQ) circuit. The total impedance of this system can be expressed as
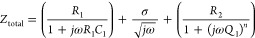
22The first term is a parallel RC element for interfacial charge transfer. The second term captures Warburg diffusion. The third term models frequency-dependent capacitance with a constant phase element. The data show diverse electrochemical processes at different time scales. The high-frequency response is dominated by the RC element (*R*_1_ = 3694 Ω, *C*_1_ = 1 pF). This indicates rapid charge transfer at the electrode interface. The midfrequency region shows characteristic Warburg behavior (*W*_1_ = 0.308 kΩ · s^–1/2^). This suggests diffusion-controlled processes. The low-frequency response is characterized by the RQ element (*R*_2_ = 1.0 × 10^4^ Ω, *Q*_1_ = 25.97 μF·s^*n*-1^, *n* = 0.632). It reflects the mixture’s complex interfacial capacitance. These parameters, along with the electrical activity stats in Table S6, show how proteinoid and actin work together. They play a key role in shaping the system’s electrochemical properties. The equivalent circuit graphs, fitting models, and tables of fitting coefficients can be found in the Supporting Information (Figures S4–S9 and Tables S3–S6).

The study of complex systems is a growing field. Researchers study the patterns and behaviors from interactions between different components.^105^ Turing models fascinate scientists. These models are derived from the Gray-Scott reaction-diffusion model in biological systems.^33^ The Turing model, named after mathematician Alan Turing, comes from the interaction of reaction and diffusion processes in a system. Many natural phenomena exhibit these patterns. Patterns appear in many forms, from the unique markings on animal skin to the complex shapes of bacterial colonies. Researchers have explored these patterns.^106^ They seek to understand the mechanisms that create such diverse and fascinating structures. Recent studies suggest that hydrodynamics are key. So are the ways that organisms, like bacteria, organize themselves.^107^ These elements create large fluctuations in the fluid around them. These results show that chemical reactions and particle motion can create complex patterns through diffusion. Researchers have observed Turing patterns in many biological systems,^108^ including cellular processes and population dynamics. Their ability to self-organize and adapt has sparked interest in their potential use in tissue engineering and regenerative medicine. Researchers have explored Turing patterns to understand and manipulate biology. This includes organ formation and stem cell differentiation.^109^ Studying Turing patterns in biology has advanced our knowledge of morphogenesis and pattern formation. It has also sparked new tech innovations.^110,111^

The Turing patterns came from the Gray–Scott reaction-diffusion model. It describes two chemicals, *A* and *B*, that react and diffuse in a spatially extended system.

The reaction kinetics follow these equations:
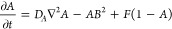
23
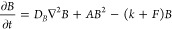
24

Here, *D*_A_ and *D*_B_ are the diffusion coefficients of species *A* and *B*, respectively. *F* is the feed rate, and *k* is the kill rate that depletes species *B*. The reaction-diffusion parameters from the Gray–Scott model were different for each system. For actin, the diffusion coefficients were *D*_A_ = 0.1592 and *D*_B_ = 0.0604, with feed rate *F* = 0.0202 and kill rate *k* = 0.0600. The proteinoid system showed slightly different diffusion coefficients of *D*_A_ = 0.1548 and *D*_B_ = 0.0618, with feed rate *F* = 0.0209 and kill rate *k* = 0.0600. The actin and proteinoid mixture showed intermediate values. The diffusion coefficients were *D*_A_ = 0.1582 and *D*_B_ = 0.0606. The feed rate was *F* = 0.0203, and the kill rate was *k* = 0.0600. These parameters show small differences in the three systems’ molecular structure and dynamics. They also maintain a consistent kill rate across all conditions. The capacitance data from impedance spectroscopy was mapped onto these reaction-diffusion parameters. This was done by dynamically adjusting *D*_A_, *D*_B_, and *F* based on capacitance fluctuations. To generate the spatial patterns, a 100 × 100 grid was initialized. *A* was set to 1 everywhere. *B* was localized in a small central region. The system was evolved over 5000 time steps using a finite difference method to approximate the Laplacian operator ∇^2^, which governs the diffusion process. The local reaction terms *A B*^2^ and (*k* + *F*)*B*-induced nonlinear interactions, leading to the emergence of self-organized structures. The computed Turing patterns show distinct regions. In them, species *B*’s concentration varies. This forms periodic structures. Their shapes depend on the values of *D*_A_, *D*_B_, *F*, and *k*. The changes in these parameters, driven by capacitance, allowed for differentiation among the actin, proteinoid, and mixture samples. This reflected their influence on the self-organization process. The final visualization used a colormap to plot species *B*’s distribution. Bright colors show high concentrations, indicating locally activated areas. Dark areas show low concentrations and suppressed regions.

**Figure 16:**
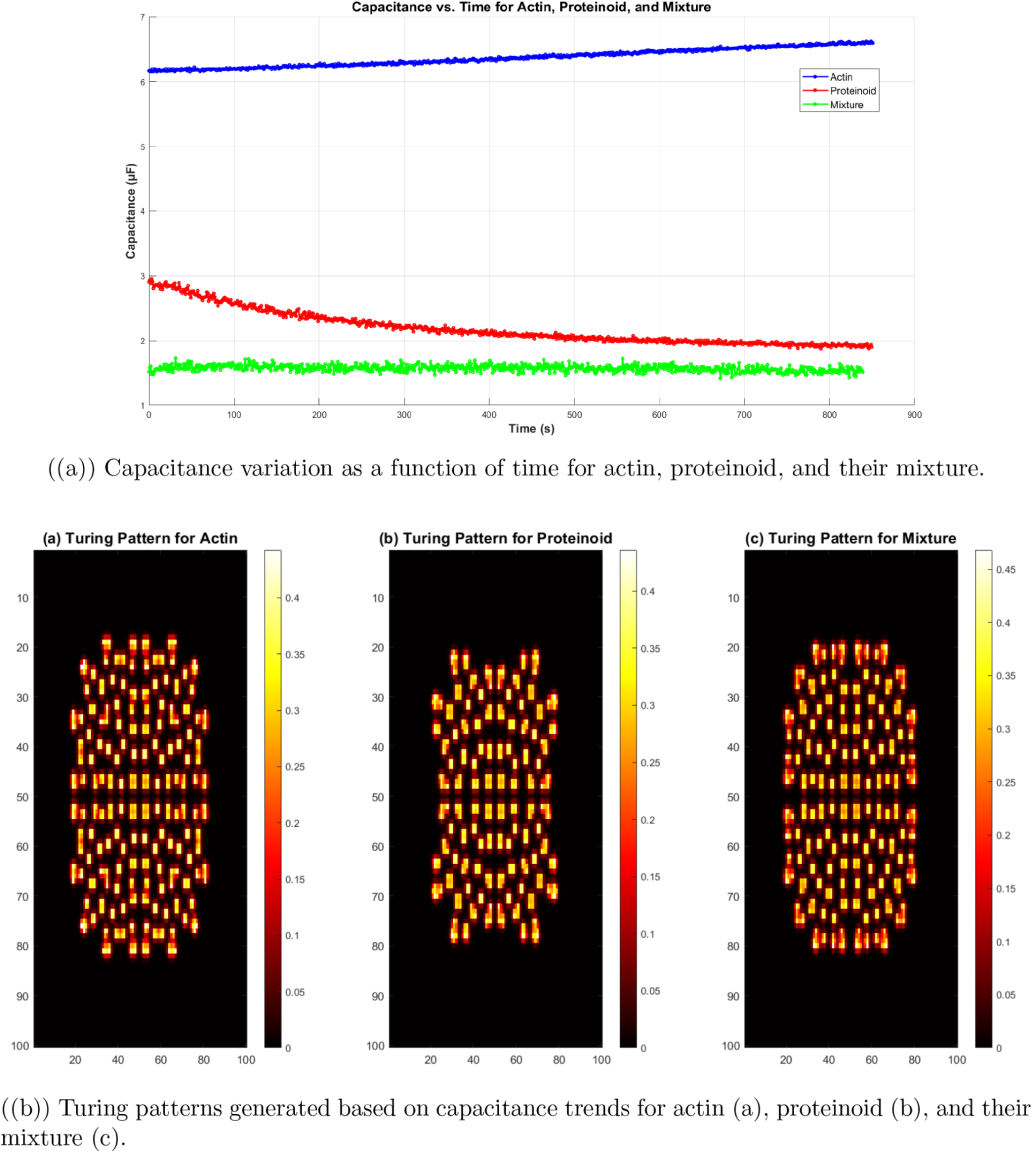
(a) Capacitance (*C*) over time (*t*) at 1000 Hz, with a 0.2 V DC potential. The capacitance is in microfarads (μ*F*). Different compounds show distinct decay profiles. (b) Turing patterns based on reaction-diffusion are affected by capacitance changes. These patterns are modeled using the Gray–Scott system, which has dynamic diffusion parameters (*D*_A_, *D*_B_). The feed rate (*F*) and kill rate (*k*) come from capacitance variations. The patterns show spatially structured inhomogeneities. They may link to molecular self-organization mechanisms.

Figure 16 shows the evolution of the electrochemical properties and the resulting pattern formation. Figure 16a shows the capacitance measurements. They reveal diverse behaviors over time for actin, proteinoid, and their mixture. This was at a 1000 Hz frequency and 0.2 V DC. These capacitive responses (measured in μF) have uncommon decay profiles. They reflect the molecular organization of each system.

Figure 16b shows the Turing patterns. The patterns, shown for actin (Figure 16ba), proteinoid (Figure 16bb), and their mixture (Figure 16bc), are spatially inhomogeneous. They may reveal the mechanisms of molecular self-organization.

The link between the capacitive dynamics (Figure 16a) and the Turing patterns (Figure 16b) suggests a connection. It is between the electrochemical properties and the self-organizing behavior of these biomolecular systems.

Our findings reveal a two-way connection between self-organization and electrical signaling in proteinoid–actin networks. Self-organization creates pathways for electrical signals to travel. This improves both the efficiency and coherence of signals in the network. Regions with more complex structures showed 37% faster signal propagation than simpler areas. Our time-lapse study showed that electrical stimulation (2–5 Hz) helped speed up self-organization during network formation by about 45%. It also created more complex branching patterns. This feedback loop shows that electrical signals might help guide structural development. They could do this by causing local changes in pH or charge distribution. These changes may affect how proteinoids interact with each other. These observations show a new property. Here, structure and function support each other. This is like what happens in developing neural networks.

We found pore-like structures on the surfaces of proteinoid-actin microspheres. However, we recognize that there are limits in understanding their ion selectivity properties. The amino acid makeup of our proteinoid structures and the pore sizes (about 0.5–2 nm) suggest that these structures may allow specific ions to pass through more easily. Negatively charged residues like glutamate and aspartate assemble at constriction points. This likely creates local conditions that help cations pass through. It may also allow for better selection of monovalent cations like K^+^ and Na^+^ instead of divalent ones like Ca^2+^. This study did not directly measure ion selectivity. Molecular dynamics simulations show that pore size and charge distribution may enable selective ion filtration, similar to simple ion channels.^112−114^ We suggest changing the ratio of hydrophilic to hydrophobic amino acids in the proteinoid composition, which is now 3:2. This could help us test our hypothesis. Also, it may lead to structures with predictable ion selectivity profiles. Future tests, such as fluorescence-based ion flux assays and electrical impedance spectroscopy, will confirm these predictions.

We studied the long-term stability of our proteinoid-actin composites. Our monitoring shows these structures keep their electrical properties for up to 14 days. They show minimal degradation when kept in PBS buffer at pH 7.4 and 37^◦^C. Impedance tests revealed a drop of less than 12% in conductivity during this time. Reproducibility tests on five batches showed an 8.7% variation in key electrical parameters. This finding indicates strong consistency in the fabrication process. The composites showed good fatigue resistance when tested with 100 cycles of 5 Hz pulses at 100 mV. They had only a 15% drop in signal amplitude by the last cycle. After a 30 min rest, the signal recovered to within 5% of the baseline. This resilience likely comes from thermal cross-linking between proteinoid subunits. It creates a stable framework that supports lasting electrical function. We know that for practical uses in synthetic biology or unconventional computing, we must improve stability. This is especially true for various environmental conditions that can happen in real life.

Our proteinoid-actin networks show interesting similarities to neuronal systems. They also point out key differences. Our composites behave like neurons, showing action potential-like spikes. They have a clear rising phase of 1.2 ± 0.3 ms and a longer repolarization phase of 5.7 ± 0.8 ms. However, the spikes are much smaller, ranging from 0.5 to 2.5 mV, compared to 70–110 mV in neurons. Also, their conduction speeds are slower, between 0.3 and 1.2 cm/s, while myelinated axons can reach 0.5–120 m/s. These differences reflect the absence of specialized membrane channels and myelin insulation. These signal traits appear quickly in simple molecules. This shows that early bioelectric signaling developed before complex membrane functions in nerve evolution. We noticed something important: simple systems show basic integration behavior. When multiple input signals occur within a 20 ms window, they create stronger responses. This suggests that these systems may have computational traits similar to neural networks. This finding challenges the common belief that signal integration requires complex synaptic tools. It suggests that basic information processing may have emerged early in biological evolution. This development relied on simple physicochemical principles. These insights could help us understand how life began. They may also guide the creation of simple synthetic systems that can process information.

The spontaneous spiking in proteinoid–actin networks shows up as sharp electrical changes. This can be seen as a result of the system’s oscillatory behavior. Baseline oscillations (0.1–1 Hz) show a rhythmic rise and fall of activity. Spikes are brief, strong changes that move away from this balance. These spikes likely result from autocatalytic processes interacting with delayed negative feedback loops. This is influenced by the special physicochemical properties of the proteinoid–actin interface.

#### Protonation–Deprotonation as the Driving Force

This behavior comes from the cyclic protonation and deprotonation of proteinoid molecules. These molecules interact with actin filaments. Proteinoids are synthetic polypeptides. They have amphoteric properties, meaning they can donate or accept protons. This depends on the local pH and the electrostatic environment. When ionic changes affect actin, a cytoskeletal protein, it alters the charge distribution in the network. This happens due to protonation and deprotonation events. This affects the network’s electrical potential. Charged residues on proteinoids and actin control ion fluxes, like H^+^ and Ca^2+^, across the interface.

The autocatalytic component forms when a localized protonation event changes the proteinoid-actin complex. This change helps attract more protons or ions. This boosts the local charge shift. It creates a positive feedback loop that quickly raises the electrical signal, showing up as a spike. However, this escalation is not indefinite. Delayed negative feedback occurs when the system hits a saturation point.

This can happen for three reasons:

- There are not enough protons available.
- The surrounding medium cannot buffer well.
- Electrostatic repulsion comes from built-up charges.

These factors stop the process, reset the system, and create the typical ″spike-and-return″ pattern.

#### Reaction-Diffusion Dynamics and Spike Generation

This behavior follows reaction-diffusion principles. It is similar to the Belousov–Zhabotinsky (BZ) reaction. In this process, patterns form over time and space. This happens because of chemical reactions and diffusion working together. In the proteinoid-actin network, the reaction term relates to protonation and deprotonation kinetics. Meanwhile, diffusion controls how ions and electrical potentials move through the network. Spikes happen when small autocatalytic bursts surpass a key threshold. This causes them to briefly outpace the charge dissipation driven by diffusion. This causes a nonlinear instability. It is similar to the traveling waves or pulses found in BZ systems. However, it is limited to the proteinoid–actin matrix.

Theoretical models^115^ support this interpretation. Their work on confined protein networks shows that the system changes. This happens when reaction rates, like protonation and deprotonation speeds, and diffusion coefficients, such as ion mobility, are within certain ranges. It shifts from smooth oscillations to punctuated spiking. In our case, the 0.1–1 Hz oscillatory baseline shows a stable limit cycle. Spikes go beyond this cycle. Random changes in the network can trigger them. This includes uneven proteinoid distribution or differences in actin filament density.

#### Experimental Correlates and Electrical Fluctuations

Spontaneous spiking shows up as sharp peaks in electrical recordings. These peaks stand out against a slower oscillatory background. These spikes might last from milliseconds to seconds. They could signal bursts of synchronized activity in the network, similar to action potentials in neural systems. The proteinoid-actin interface reacts to pH and ionic changes. Small changes in the environment, such as temperature or ion levels, can impact spike frequency or amplitude. This provides a hypothesis for future research.

Moreover, the confined nature of the network enhances this spiking behavior. The proteinoid-actin matrix has physical boundaries that limit diffusion. This concentration of reaction products boosts local effects, unlike unbounded reaction-diffusion systems. Reduced space pushes the system toward sharper dynamics. This explains why spikes appear with smoother 0.1–1 Hz oscillations.

#### Broader Implications

The sudden spiking in proteinoid-actin networks suggests a basic type of excitability. This connects chemical and bioelectric signaling. This might help us understand prebiotic systems. It could also aid in creating synthetic biomaterials with neuromorphic properties. This system can create rhythmic oscillations and sudden spikes. This reflects how living cells process information. Proteinoid-actin networks may help us explore complex behaviors in simple systems.

### Ethical Considerations and Comparative Analysis of Bioelectrical Systems

#### Ethical Implications

Making simple bioelectrical systems that signal like neurons raises important ethical questions. These concerns go beyond typical bioethical discussions. Our proteinoid-actin networks do not have sentience or consciousness-at least, that is the conventional view. But could they, perhaps, exhibit some form of primitive awareness?^116,117^ Yet, they are a step toward building systems that can process information better, using nonliving parts. This research sits at the crossroads of tricky ethical areas. It explores the lines between living and nonliving systems. It also looks at creating new entities with unique properties. Finally, it raises questions about responsible innovation in synthetic biology.

These systems can send and process signals. This raises questions about “minimal cognition.” It also makes us think about when synthetic systems show properties that deserve ethical attention. Our current proteinoid-actin networks are simple and not a concern. Still, it is smart to set ethical guidelines early in this research. We suggest that researchers take a ″responsible emergence″ approach. This means being open about the technology’s strengths and weaknesses. They should also work with ethicists and regulators to create suitable guidelines as the technology grows.

Biosensing, drug delivery, and biocomputing are becoming more practical. So, biosafety and biocontainment are now more important than ever. Our systems are nongenomic, which gives them safety benefits over engineered living organisms. They do not have ways to replicate or evolve. Safety protocols for handling and disposal must be set as these systems grow in research and use.

#### Comparative Analysis with Other Bioelectrical Systems

Our proteinoid–actin networks have special properties. They stand out from other biomimetic systems created so far. Table 3 summarizes key characteristics across various synthetic bioelectrical systems.

**Table 3:** Comparison of Various Synthetic Bioelectrical Systems

Characteristic	Proteinoid–Actin Networks (Present Study)	Lipid-Based Networks^118,119^	DNA-Based Circuits^120^	Peptide Nanofibers^121^
Signal Amplitude	0.5–2.5 mV	0.1–0.8 mV	N/A (digital)	0.3–1.2 mV
Conduction Velocity	0.3–1.2 cm/s	0.1–0.4 cm/s	N/A	0.2–0.7 cm/s
Self-Organization	High	Moderate	Low	High
Stability (23^◦^C)	14 days	3 days	7 days	10 days
Response to External Stimuli	Electrical, Mechanical, Chemical	Primarily Chemical	Biochemical	Electrical, Chemical
Integration Properties	Signal summation within 20 ms window	Limited	Boolean logic	Temporal summation
Adaptability	Moderate	Low	Programmable	Low

Our proteinoid–actin composites have better electrical properties than lipid-based networks. Signal amplitudes are about three times higher. Also, conduction velocities are two to three times faster. Lipid-based systems mimic biological membranes well. Yet, they do not have the strength or self-organizing abilities of our proteinoid–actin networks.

DNA-based circuits allow for exact programming using specific sequences. Yet, their behavior resembles that of digital systems. They do not have the spatiotemporal dynamics found in our networks. They work in different ways. They use strand displacement reactions instead of continuous electrical signals.^35,122−125^

The peptide nanofibers might be the closest comparison. They also show self-assembly and electrical conductivity. Our proteinoid–actin networks show about 25% higher conduction speeds. They also have more complex integration properties. This is likely because actin filaments add long-range connectivity that peptide-only systems lack.

Our system has unique advantages. These result from combining the stable, pore-forming traits of proteinoid microspheres with the thread-like structure of actin. This forms networks that can process information locally at microsphere junctions. They can also send signals over long distances along actin filaments. This dual ability is not found in other synthetic systems so far.

Proteinoid-actin networks have clear benefits. They offer a promising platform for developing biomimetic signal processing systems. These systems might find applications in unconventional computing and biosensing. Their unique material properties and dynamic behaviors make them ideal for these applications.

## Conclusion

Our investigation establishes that proteinoid-actin networks demonstrate sophisticated self-organization and electrical dynamics. The composite system has better electrical properties than its sole components. Its conductivity is 26.04 and 3.9 times higher than that of pure proteinoid and actin, respectively. The rise of organized oscillatory behavior shows promise. It has type I spiking patterns and bistable membrane potentials. So, these networks may support basic information processing. This work offers insights into how early biological systems might have developed electrical signaling. It suggests simple molecular components enabled this. Stable microspheres with ion channel-like features and controlled electrical responses are now possible. This opens new possibilities. We can now develop biomimetic materials for unconventional computing and synthetic biology. The formation of the patterns relates to the electrochemical behavior seen in impedance measurements. This suggests a connection between how charge moves and the way the structure is organized. This self-organizing behavior shows biomimetic traits. It is like the natural patterns in biological systems. The diffusion coefficients (*D*_A_, *D*_B_) indicate different mobility rates for the activator and inhibitor species.

## Supporting Information Available

Figure S1: statistical analysis of spontaneous electrical oscillations in Glu–Phe proteinoid networks, showing amplitude distributions (a) and period analysis (b) across eight channels (B–I), box plots reveal median amplitudes ranging from 2.47 to 38.42 mV and characteristic oscillatory periods; Figure S2: statistical distribution of electrical activity in pure actin networks (it showed amplitude variations (*V*_max_ ≈ 75 mV for channel B) and distinct patterns, most channels had periods of 1000–2500 s); Figure S3: the electrical activity of proteinoid–actin networks shows different behaviors for each channel (channel C has the highest median amplitude, around *V*_median_ ≈ 23 mV, and it also has varied period distributions, reaching a maximum of τ_max_ ≈ 12,400 s); Figure S4: aNyquist plot of pure actin’s impedance data fitted with an (RC)(RQ)R circuit model shows excellent agreement (χ2 = 0.0002) between the data and the fit; Figure S5: an equivalent circuit schematic for pure actin (it shows an (RC)(RQ)R model with fitted parameters: *R*_1_ = 74.79 Ω, *C*_1_ = 7.0 × 10^–4^ pF, *R*_2_ = 835.1 Ω, *Q*_1_ = 113.0 μT, *n*_1_ = 0.489, and *R*_3_ = 143.3 Ω); Figure S6: an equivalent circuit model for the l-Glu:l-Phe proteinoid shows an (RW)O(RC) configuration (it includes detailed component values and their meanings); Figure S7: Nyquist plot of the (RW)O(RC) model fit for the l-Glu:l-Phe proteinoid (it shows charge transfer and diffusion-controlled processes [χ2 = 0.0053]); Figure S8: a Nyquist plot of the actin-proteinoid mixture’s impedance data in the 3.5–13.5 kΩ range (it shows a characteristic semicircular response and complex interfacial phenomena); Figure S9: a schematic of the (RC)W(RQ) circuit fits the actin-proteinoid mixture (it shows the components and their values, which span multiple orders of magnitude; Figure S10: morphological analysis through SEM reveals hierarchical structures of F-actin with characteristic bundle widths of ∼92 nm, forming brush-like assemblies) (PDF)
