# Supplementary material for: Self-Organizing Proteinoid–Actin Networks: Structure and Voltage Dynamics
Source: ACS Omega. 2025 May 5;10(18):18986–9009. doi: 10.1021/acsomega.5c01141 (PMC12079275; doi:10.1021/acsomega.5c01141)
Supplement: Supplementary file 1 — ao5c01141_si_001.pdf [file ao5c01141_si_001.pdf]

Supporting Information

Self-Organizing Proteinoid-Actin Networks: Structure and Voltage Dynamics

Panagiotis Mougkogiannis[1,*] and Andrew Adamatzky[1]

[1]Unconventional Computing Laboratory, University of the West of England, Bristol, UK, BS16 1QY

Email: Panagiotis.Mougkogiannis@uwe.ac.uk

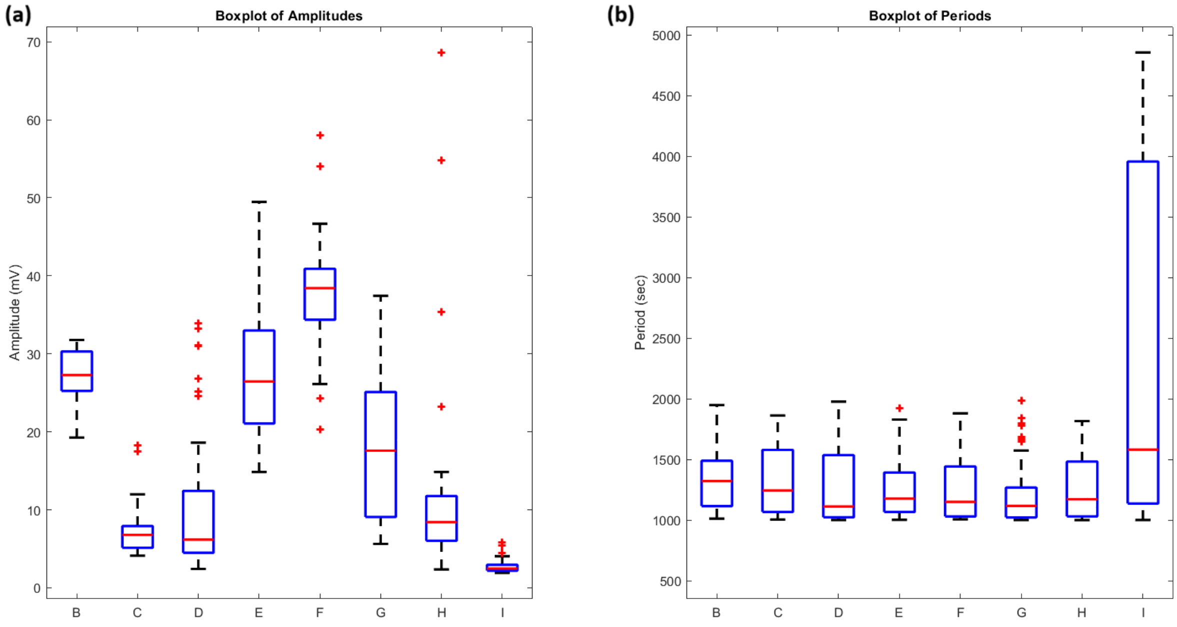

Figure S1: Statistical characterization of spontaneous electrical oscillations in Glu-Phe proteinoid networks. (a) A box plot of amplitude distributions across eight channels (B-I) shows median amplitudes of 2.47 mV (Channel I) to 38.42 mV (Channel F). Channel F has the highest stable amplitude (median = 38.42 ± 7.24 mV) and Channel I the lowest (median = 2.47 ± 1.14 mV). Outliers (red crosses) indicate occasional high-amplitude events, particularly in Channel H (max = 68.63 mV). (b) Period analysis shows consistent oscillatory behavior in channels B-H (median periods 1100-1600 s). Channel I has longer periods (median = 1580.55 s, max = 4855.45 s). The standard deviations of channels B-H (255-321 s) are stable. In contrast, Channel I's is 1427.39 s. This suggests different mechanisms for generating oscillations.

Table S1: Quantitative Analysis of Spontaneous Electrical Activity in Glu-Phe Proteinoid Networks.

Channel	Amplitude (mV)				Period (s)			
	Median	σ	Min	Max	Median	σ	Min	Max
B	27.27	3.39	19.26	31.77	1321.80	267.83	1011.55	1947.75
C	6.79	3.31	4.13	18.26	1243.62	269.79	1003.70	1862.20
D	6.18	9.95	2.42	33.90	1111.75	320.81	1000.35	1976.45
E	26.45	9.26	14.85	49.48	1176.90	255.06	1002.40	1919.80
F	38.42	7.24	20.30	57.98	1149.85	268.69	1006.20	1880.00
G	17.58	8.93	5.63	37.44	1116.30	292.84	1000.35	1983.35
H	8.42	13.65	2.36	68.63	1171.90	264.61	1000.10	1816.10
I	2.47	1.14	1.92	5.84	1580.55	1427.39	1000.45	4855.45

Note: Statistical analysis of spontaneous electrical oscillations across eight channels (B-I). It shows their amplitude and timing. Amplitude measurements reveal distinct activity patterns. They range from high-amplitude stable oscillations (Channel F) to low-amplitude fluctuations (Channel I). Period analysis shows consistent oscillatory behavior in channels B-H ($\sigma \approx$ 250-320 s). Channel I has a broader temporal distribution (σ = 1427.39 s). The regular changes in amplitude and period suggest mixed but organized electrical activity across the proteinoid-actin network.

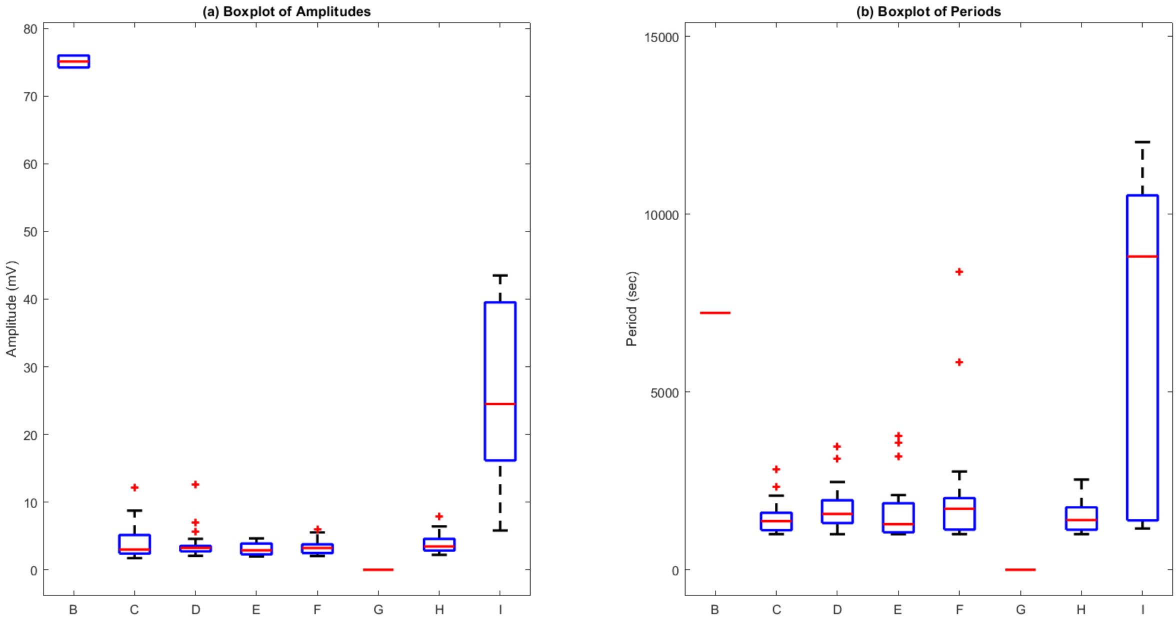

Figure S2: Statistical distribution of electrical activity parameters in pure actin networks. A boxplot of amplitude variations across channels B-I. It shows the median, quartiles, and outliers. Channel B has high amplitudes (~75 mV). Channel I has a broad amplitude distribution ($V_{\text{median}} \approx 24.5$ mV) with high variability. Channels C-H demonstrate lower amplitude responses (2-4 mV) with occasional outliers. (b) Period distribution analysis revealing distinct temporal patterns. Most channels (C-H) have consistent periods (1000-2500 s). Channel I has a median period of $\tau_{\text{median}} \approx 8800$ s. Its periods vary widely. Channel B shows stable periodic behavior ($\tau \approx 7225$ s). Red crosses (+) indicate statistical outliers. The boxplots show the interquartile range (IQR, blue boxes), the median (red line), and the whiskers. They extend to the most extreme non-outlier values (±1.5 IQR).

Table S2: Quantitative Analysis of Electrical Activity in Pure Actin Networks. The data shows unique electrical patterns across channels. Channel B has high-amplitude stable activity ($V_{\max} \approx 76$ mV). Other channels have varying electrical responses. Channels F and I show notable time variations. This suggests complex actin-membrane interactions with irregular electrical responses.

**Channel**	**Amplitude (mV)**				**Period (s)**			
	Mean	σ	Min	Max	Mean	σ	Min	Max
B	75.12	1.25	74.24	76.00	7225.05	0.00	7225.05	7225.05
C	4.03	2.38	1.71	12.20	1457.02	425.79	1001.20	2817.45
D	3.70	2.09	2.06	12.56	1724.66	611.68	1000.40	3459.00
E	3.07	0.84	1.96	4.64	1587.10	769.16	1000.05	3766.40
F	3.36	1.14	2.03	6.00	2131.48	1768.87	1001.20	8386.65
G	0.00	0.00	0.00	0.00	0.00	0.00	0.00	0.00
H	3.81	1.26	2.20	7.85	1517.81	485.07	1000.90	2537.75
I	26.29	14.09	5.80	43.48	7121.08	4742.22	1159.30	12028.70

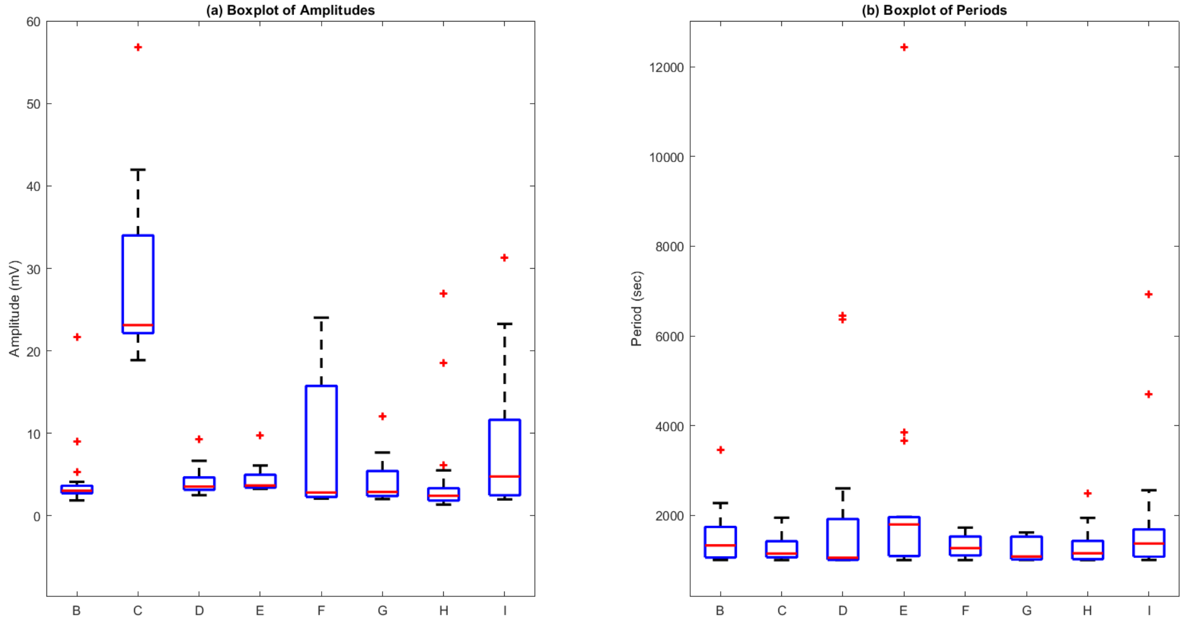

Figure S3: Statistical distribution of electrical activity parameters in proteinoid-actin networks. Amplitude analysis reveals distinct channel-specific behaviours. Channel C has the highest median amplitude ($V_{\text{median}} \approx 23$ mV) and high variability (IQR: 22.16-34.00 mV). Channels F and I have moderate amplitudes with wider distributions ($\sigma_F = 9.64$ mV, $\sigma_I = 7.88$ mV). Other channels show low-amplitude responses ($V_{\text{median}} \approx$ 2-4 mV). (b) Period distribution analysis shows patterns over time. Most channels have periods of 1000-2000 s. Channel E displays extended periods with significant outliers, with $\tau_{\max}$ approaching 12400 s. Red crosses (+) indicate statistical outliers beyond 1.5×IQR. The boxplots show the IQR (blue boxes), median (red line), and whiskers that extend to the most extreme non-outlier values. The varied amplitude and period distributions suggest complex patterns in the proteinoid-actin network's dynamics.

Table S3: Quantitative Analysis of Electrical Activity in Proteinoid-Actin Networks. The data reveals complex electrical patterns with Channel C showing highest mean amplitude ($V_{mean} \approx 27.66$ mV). Temporal analysis indicates varied periodicities, with Channel E exhibiting extended periods ($\tau_{max} \approx 12441$ s). The varied amplitudes and timing suggest complex interactions. They are between the proteinoid and actin components. These interactions cause coordinated electrical responses.

**Channel**	**Amplitude (mV)**				**Period (s)**			
	Mean	σ	Min	Max	Mean	σ	Min	Max
B	3.88	3.48	1.87	21.70	1458.82	534.29	1001.80	3457.00
C	27.66	8.66	18.88	56.84	1256.47	262.59	1000.85	1944.15
D	4.17	1.50	2.50	9.29	1750.54	1484.64	1000.05	6457.95
E	4.40	1.66	3.27	9.76	2502.96	2885.95	1001.05	12441.45
F	8.86	9.64	2.10	24.03	1315.00	304.06	1001.05	1723.60
G	4.40	3.36	2.04	12.02	1231.91	274.00	1002.50	1616.60
H	3.86	4.98	1.37	26.96	1285.58	351.43	1000.10	2486.00
I	8.54	7.88	1.99	31.33	1749.13	1324.79	1002.85	6922.85

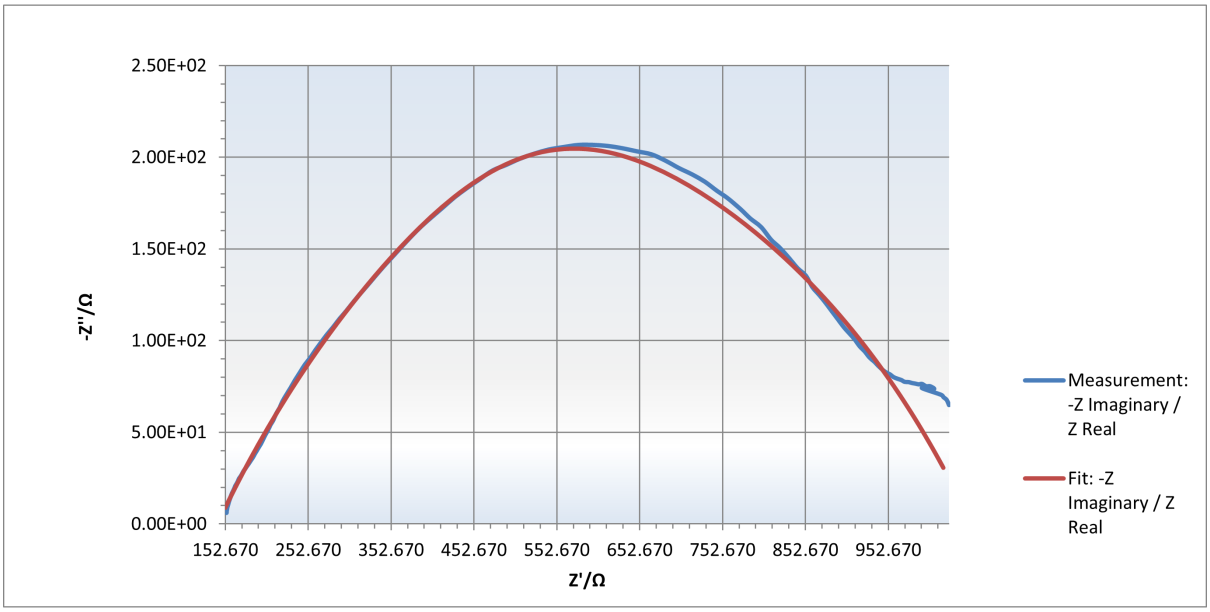

Figure S4: A Nyquist plot showing the experimental impedance data (blue line) and a theoretical fit (red line) for pure actin using an (RC)(RQ)R circuit model. The measured and fitted data match well ($\chi^2 = 0.0002$). This validates the circuit model. It shows a semicircular response, as expected from the parallel RC and RQ elements. The x-axis represents the real impedance (Z') and the y-axis shows the negative imaginary impedance ($-Z''$) in ohms (Ω).

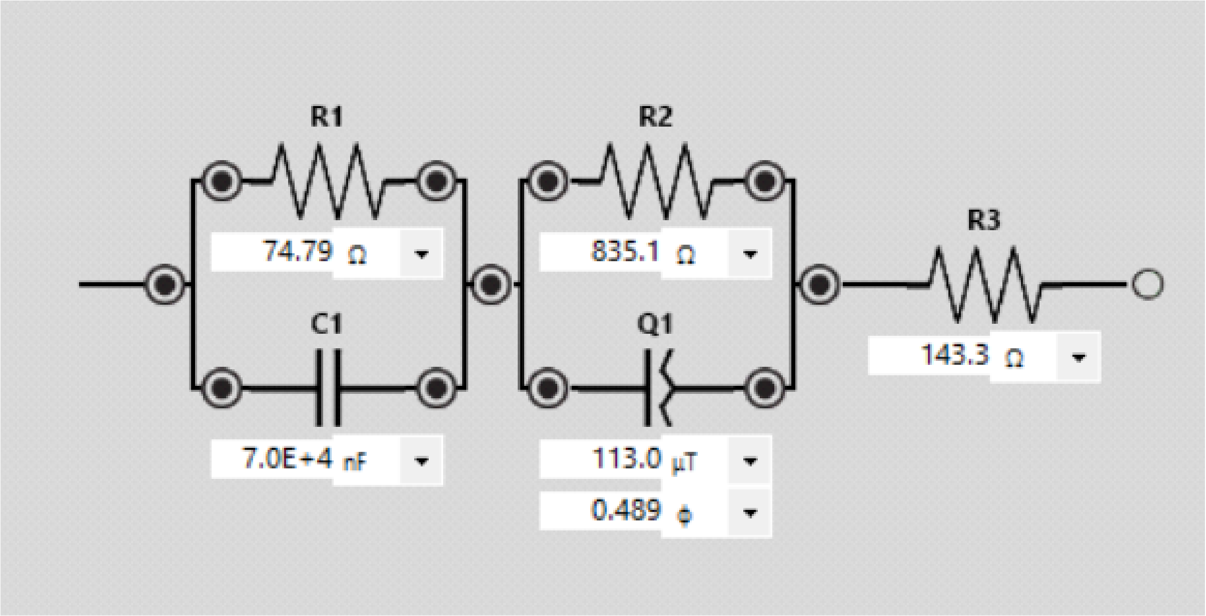

Figure S5: Equivalent circuit model used to fit the electrochemical impedance spectroscopy data of pure actin. The circuit consists of a parallel RC element ($R_1 = 74.79\ \Omega$, $C_1 = 7.0 \times 10^{-4}$pF) in series with a parallel RQ element ($R_2 = 835.1\Omega$, $Q_1 = 113.0\ \mu$T, $n_1 = 0.489$) and a series resistor ($R_3 = 143.3\ \Omega$). This (RC)(RQ)R model provided excellent agreement with experimental data ($\chi^2 = 0.0002$).

Table S4: Fitted impedance parameters from impedance spectroscopy for the (RC)(RQ)R circuit model of pure Actin sample.

Element	Fitted Value	Min Value	Max Value	Error (%)
R_1 (Ω)	74.79	1.0×10^{-6}	1.0×10^{12}	10.65
C_1 (nF)	69.83	1.0×10^{-12}	1.0×10^{-3}	13.39
R_2 (Ω)	835.1	1.0×10^{-6}	1.0×10^{12}	0.947
Q_1 (T^{-1})	1.13×10^{-4}	1.0×10^{-12}	1.0×10^{-3}	3.807
n_1 (ϕ)	0.489	0	1.0	0.998
R_3 (Ω)	143.3	1.0×10^{-6}	1.0×10^{12}	0.520

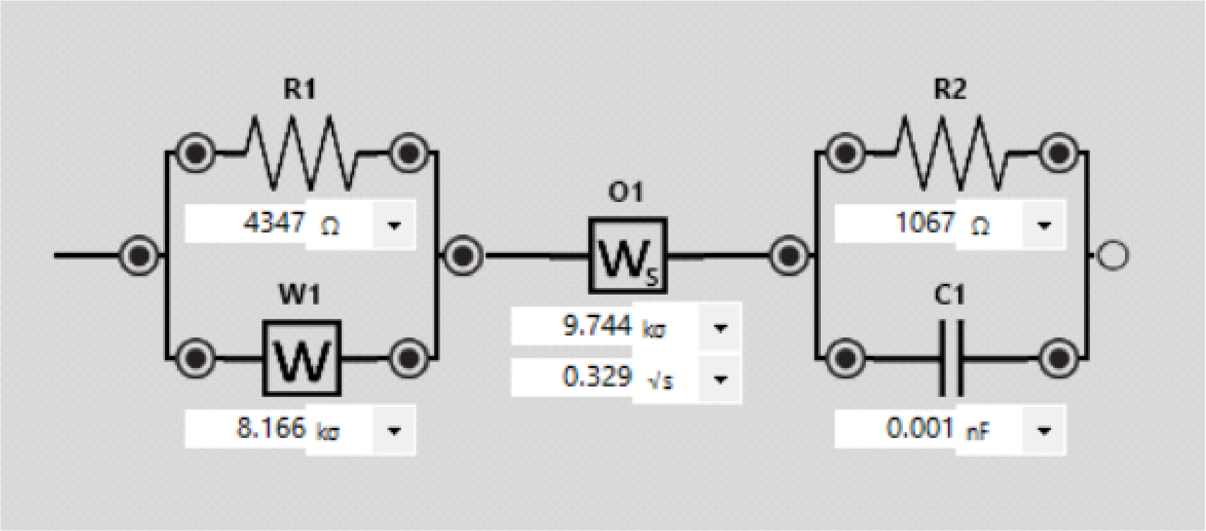

Figure S6: An equivalent circuit model for impedance analysis of the L-Glu:L-Phe sample. It has: a parallel (RW) element ($R_1 = 4347\ \Omega$, $W_1 = 8.166\ \mathrm{k\Omega \cdot s^{-1/2}}$) in series with a finite-length Warburg element ($O_1 = 9.744\ \mathrm{k\Omega \cdot s^{-1/2}}$, $\tau = 0.329\ \sqrt{\mathrm{s}}$) and a parallel RC element ($R_2 = 1067\ \Omega$, $C_1 = 1$ pF). The circuit elements represent distinct electrochemical processes: charge transfer resistance (R_1), semi-infinite diffusion (W_1), finite-length diffusion (O_1), interfacial capacitance (C_1), and secondary charge transfer resistance (R_2). This (RW)O(RC) model captures the high-frequency kinetic and low-frequency mass transport phenomena seen in the impedance spectra.

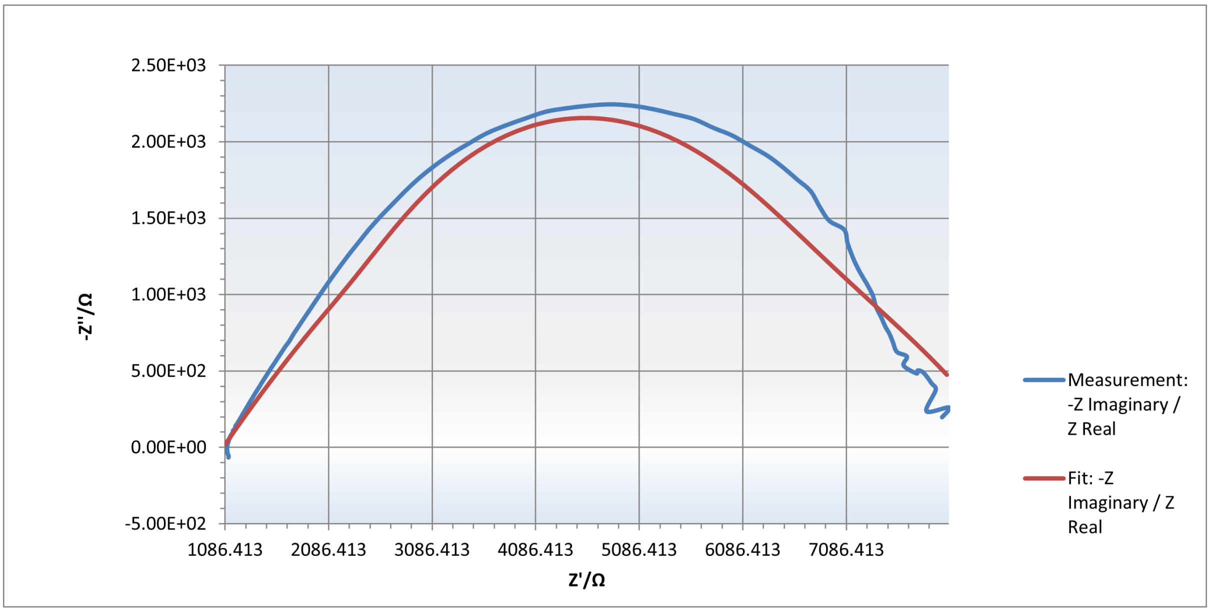

Figure S7: Nyquist plot showing the experimental impedance data (blue line) and theoretical fit (red line) for the (RW)O(RC) equivalent circuit model. The plot displays the negative imaginary impedance ($-Z''$) versus real impedance (Z') in ohms (Ω). The semicircular response with a low-frequency tail shows charge transfer and diffusion-controlled processes. The measured and fitted data match well ($\chi^2 = 0.0053$). This validates the circuit model. The high-frequency deviation (>7000 Ω) suggests additional interfacial phenomena. The 45° slope region shows the typical Warburg-like behaviour. The finite-length diffusion element (O) captures the low-frequency response transition.

Table S5: Parameters from EIS fitting of the (RW)O(RC) model. The circuit elements are resistors (R), a Warburg element (W), a finite-length diffusion element (O), and a capacitor (C). The low chi-squared value ($\chi^2 = 0.0053$) shows great agreement between the model and the experimental data after 499 iterations. The error percentages reflect the uncertainty in the fitted parameters.

**Element**	**Fitted Value**	**Unit**	**Error (%)**
R_1	4334	Ω	20.33
W_1	8224	$\Omega \cdot s^{-1/2}$	53.14
O_1	9741	$\Omega \cdot s^{-1/2}$	26.27
O_1	0.329	$\sqrt{s}$	6.16
O_1	1.000	–	15.67
R_2	1068	Ω	1.90
C_1	1.000×10^{-12}	F	52.73

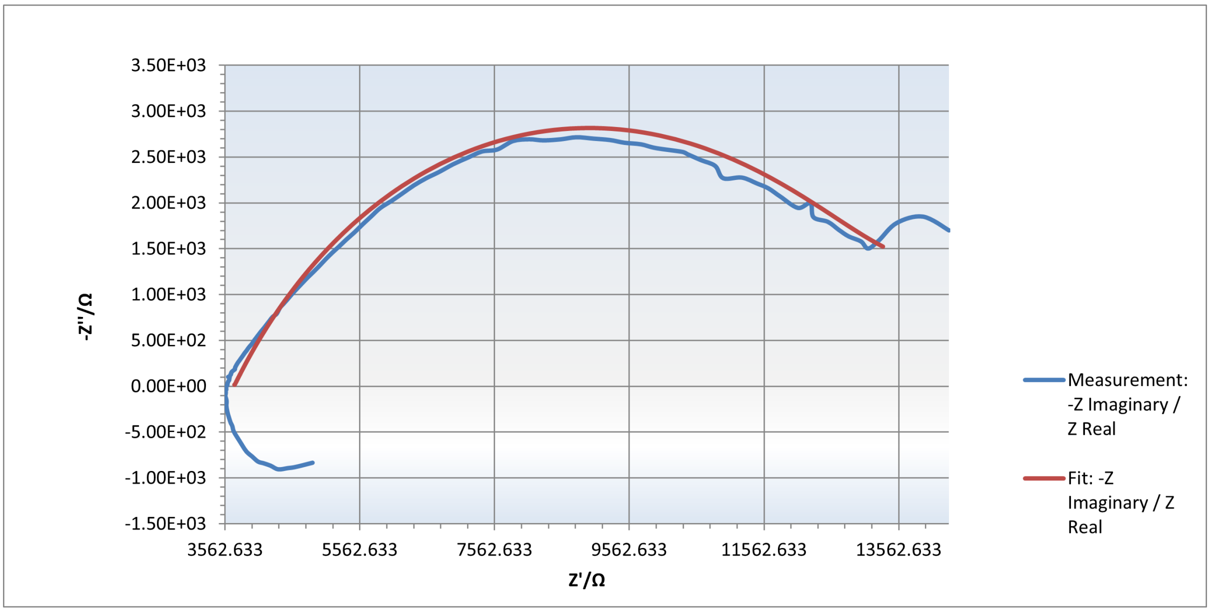

Figure S8: Nyquist plot of EIS data for the actin-proteinoid mixture. It shows both experimental measurements (blue line) and a theoretical fit (red line) in the frequency domain. The plot displays the negative imaginary impedance ($-Z''$) versus real impedance (Z') in ohms (Ω) over a range of 3.5-13.5 kΩ. The characteristic semicircular response exhibits a maximum $-Z''$ value of approximately 2.8 kΩ at $Z' \approx 9.5$ kΩ. The deviation between experimental and fitted data at high frequencies (low Z' values) and a second time constant at low frequencies (high Z' values) suggest complex interfacial phenomena. The model agrees well in the mid-frequency region. Here, charge transfer and diffusion processes dominate.

Table S6: Parameters from fitting EIS of the actin-proteinoid mixture using an (RC)W(RQ) model. The circuit incorporates parallel RC and RQ elements connected by a Warburg diffusion element (W). The model converged with $\chi^2 = 0.0067$ after 308 iterations. This shows a strong match between theoretical and experimental impedance data. The low error percentages for most elements, especially R_1 and n_1, suggest a valid model of the interfacial processes.

**Element**	**Fitted Value**	**Unit**	**Error (%)**
R_1	3694	Ω	1.21
C_1	1.000×10^{-12}	F	1909
W_1	307.7	$\Omega \cdot s^{-1/2}$	140.8
R_2	1.001×10^{4}	Ω	10.62
Q_1	2.597×10^{-5}	T	10.93
n_1	0.6320	–	4.85

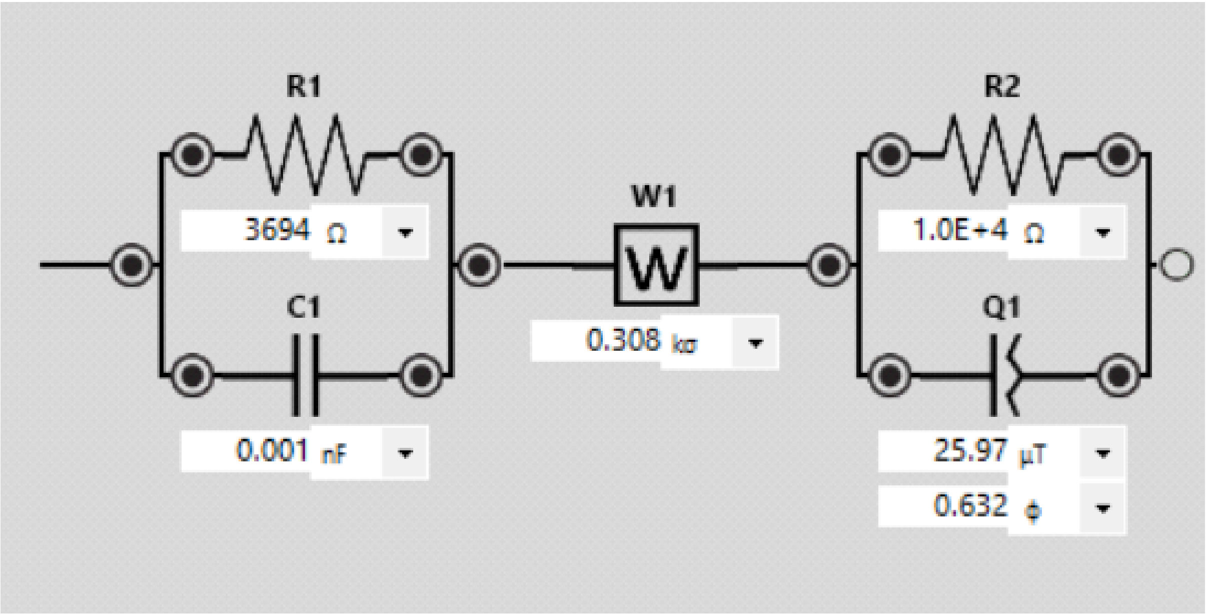

Figure S9: A schematic of the (RC)W(RQ) equivalent circuit model fitted to the actin-proteinoid mixture impedance data. The circuit has a parallel RC element ($R_1 = 3694\ \Omega$, $C_1 = 1$ pF). It represents the charge transfer resistance and double-layer capacitance. This is in series with a Warburg element ($W_1 = 0.308\ \mathrm{k}\Omega \cdot \mathrm{s}^{-1/2}$) that describes semi-infinite diffusion processes. The final component comprises a parallel combination of resistance ($R_2 = 1.0 \times 10^4\ \Omega$) and constant phase element ($Q_1 = 25.97\ \mu$T, $\phi = 0.632$). The units span several orders of magnitude. The capacitive element is in picofarads (pF). The resistive components are in kilohms (kΩ). This reflects the diverse electrochemical processes at different frequencies.

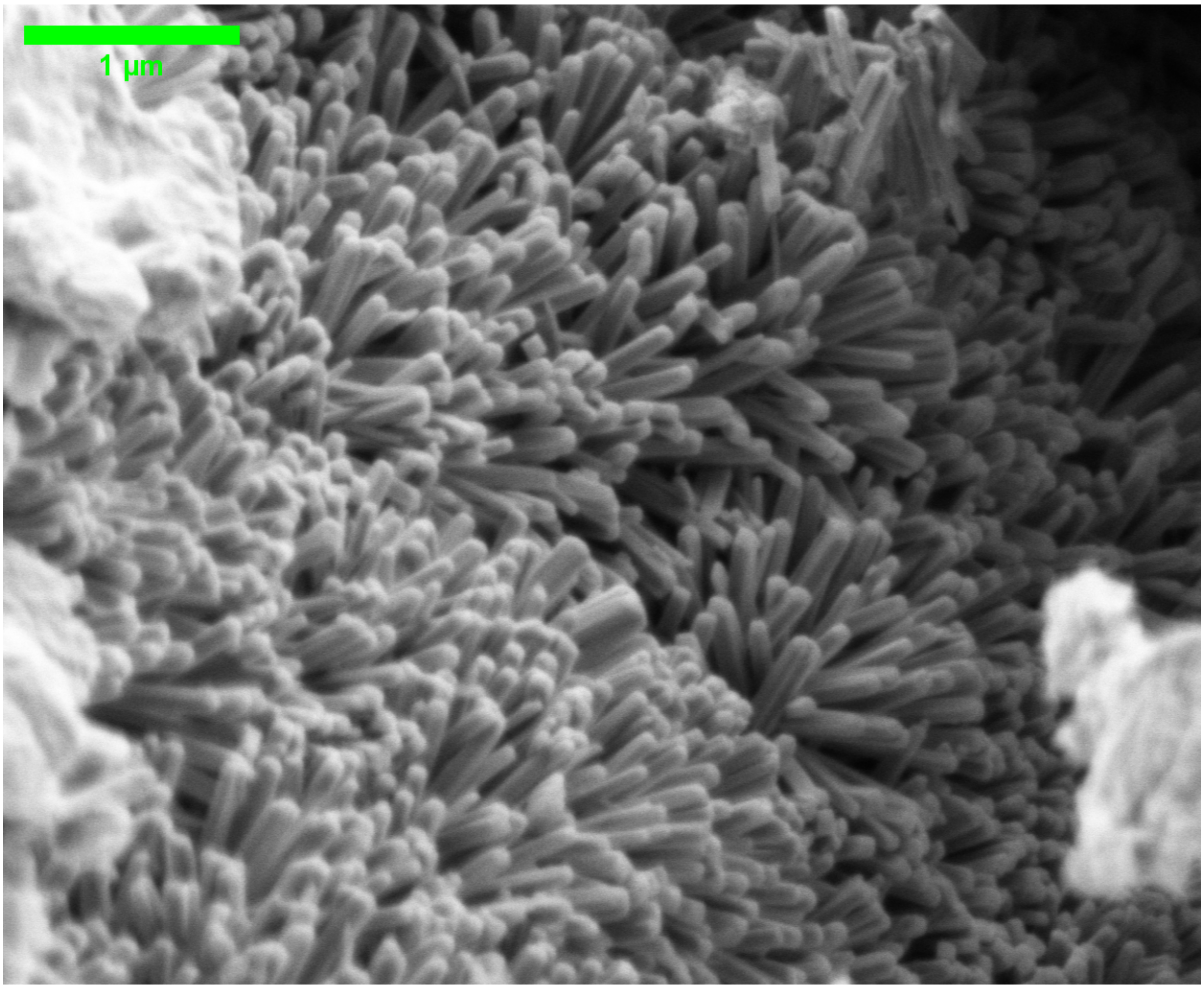

Figure S10: Scanning electron microscopy (SEM) micrograph of self-assembled F-actin filaments showing characteristic brush-like morphology. The filaments display uniform width of $\sim$92 nm arranged in hierarchical structures. Image acquired at $30,000\times$ magnification, $E_{\mathrm{acc}} = 2.00$ kV, working distance = 2.8 mm, under high vacuum (5.27×10^{-6} Torr). Scale bar: 1 μm.
